# A Chemocatalytic Route to Stereoregular Poly(3-hydroxyhexanoate) and Its Statistical and Tri-Block Copolymers

**DOI:** 10.1021/acs.biomac.6c00167

**Published:** 2026-02-23

**Authors:** Min Zhu, Maëlle T. Gace, Zhen Zhang, Eugene Y.-X. Chen

**Affiliations:** Department of Chemistry, 3447 Colorado State University, Fort Collins 80523-1872, Colorado, United States

## Abstract

Poly­(3-hydroxyhexanoate) (P3HHx)-based poly­(3-hydroxyalkanoate)­s (PHAs) are biologically produced, commercially implemented PHAs, but little is known in the open literature about the structure and property characterizations of discrete, authentic homopolymer P3HHx, and its copolymers with poly­(3-hydroxybutyrate) (P3HB). Here, we introduce a chemocatalytic route to stereoregular (both isotactic and syndiotactic) P3HHx and PHA copolymers with P3HB, including statistical copolymer P3HBHx with various levels of 3HHx incorporation and discrete hard–soft–hard triblock copolymer P3HB-*b*-P3HHx-*b*-P3HB, and present extensive characterizations of their structures, thermal properties, and mechanical performance. The synthesis is efficiently achieved by one-pot polymerization of eight-membered di­(*n*-propyl) and dimethyl (for copolymerization) diolides catalyzed by chiral molecular catalysts. Notably, P3HBHx can be rapidly produced in quantitative yield and exhibits high molar mass (*M*_n_ up to 551 kg/mol) as well as both high modulus (up to 1.39 GPa) and ductility (up to 445%), while P3HB-*b*-P3HHx-*b*-P3HB further extends application temperature windows by possessing a unique combination of low *T*_g_ (−18 °C) and high *T*_m_ (154 °C) values. These findings highlight the stereomicrostructural and architectural versatility of chemocatalytic routes to PHAs, which, in turn, can be utilized to largely tune the PHA thermal properties and mechanical performance.

## Graphical abstract

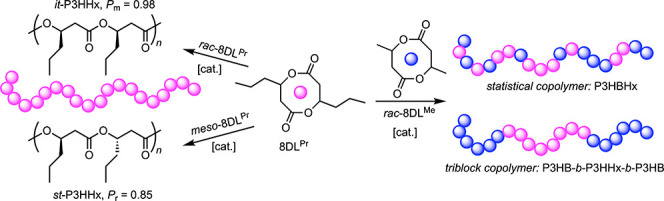

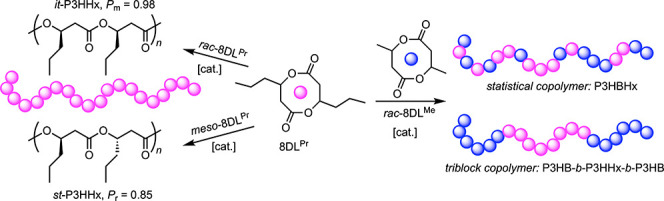

## Introduction

Worldwide plastic pollution has received widespread attention due to the persistence of nondegradable petroleum-based plastics when discarded to various natural environments after their service life.^1–4^ To address this end-of-life problem of the nondegradable plastics, intense research has focused on developing more sustainable polymers with universal biodegradability.^5–12^ In this context, poly­(3-hydroxyalkanoate)­s (PHAs), a class of aliphatic polyesters naturally accumulated by living microorganisms from biorenewable resources, are biodegradable in ambient environments and have thus drawn considerable attention.^13–22^ Within the large PHA family, the most extensively studied PHA is microbial isotactic poly­[(*R*)-3-hydroxybutyrate], P­[(*R*)-3HB], which exhibits high crystallinity with a melting temperature (*T*_m_) of 170–180 °C.^21^ However, its perfect isotacticity and high crystallinity make it extremely brittle, significantly limiting its potential for broad applications. To address this limitation, various PHA copolymers by incorporating more flexible comonomer units into the P3HB chains have been commercially developed or implemented.^23–25^ For example, both Kaneka^26^ and Bluepha^27^ developed biological routes to statistical copolymer poly­(3-hydroxybutyrate-*co*-3-hydroxyhexanoate), abbreviated here as P3HBHx, highlighting the importance and industrial relevance of poly­(3-hydroxyhexanoate) (P3HHx) and its copolymers.

Compared with biosynthetic pathways, chemosynthetic routes could offer rapid catalyst modification to adjust polymer microstructures and potential advantages in production scalability and speed.^28,29^ Early work on synthetic P3HB has focused on developing methodologies to afford biomimetic isotactic (*it*)-P3HB. The most studied approach is the ring-opening polymerization (ROP) of racemic β-butyrolactone (*rac*-BBL) to produce diverse *iso*-rich P3HB materials (isotacticity is defined here as percent meso triad [mm] and *P*_m_ as the probability for meso (RR or SS) placement of the two adjacent monomer repeating units).^30–38^ The recently developed chemocatalytic ROP of bioderived racemic eight-membered cyclic dimethyl diolide (*rac*-8DL^Me^) by yttrium complexes [Y] supported by racemic *C*_2_-symmetric salen ligands produces P3HB with perfect isotacticity ([*mm*] >99%), *P*_m_ >0.99, high *T*_m_ (∼174 °C), high number-average molar mass (*M*_n_ = 1.90 × 10^5^ g/mol), and low dispersity (*D̵* = 1.03).^39,40^ This highly stereoselective and controlled catalytic synthesis has been successfully extended to access other stereoregular PHAs.^40–42^

To overcome certain limitations of P3HB, copolymerization methods offer an effective strategy for tailoring the properties of the resulting materials by changing the comonomer or adjusting the copolymer composition.^43–45^ To address the limited processability and brittleness of *it*-P3HB, researchers have developed more flexible copolymers by incorporating comonomers with short and medium side chains from the PHA family. For example, copolymerizations of *rac*-8DL^Me^ with *rac*-8DL^R^ (*R* = ethyl, *n*-butyl, benzyl) afforded PHA statistical copolymers with various levels of 3-hydroxyalkanoate units (derived from the ROP of *rac*-8DL^R^) incorporation and thus tunable thermal and mechanical properties.^41,42^ In addition, diastereoselective polymerization enabled the direct copolymerization of diastereomeric mixtures of the same or different 8DL^R^ into stereosequenced semicrystalline stereoblock or tapered stereoblock microstructures.^40,53^ We note here that a biologically produced diblock copolymer of P3HB and P3HHx, P3HB-*b*-P3HHx (which was obtained after solvent fractionation of the isolated PHA products), and statistical copolymer P3HBHx were previously reported.^46,47^ However, the extent of P3HB-*b*-P3HHx formed in the total PHA product mixture was not reported or unknown, and mechanical tests of P3HB-*b*-P3HHx with 42 mol % 3HHx units showed a soft and weak material, with a low Young’s modulus (*E*) of only 7.6 MPa and ultimate tensile strength (σ_B_) of only 1.4 MPa.^46^

In this work, we aimed to provide synthetically authenticated P3HHx with different stereomicrostructures (in both isotactic and syndiotactic tacticities) and 3HHx-3HB copolymers with different sequences in the form of statistical copolymer and triblock copolymer (tri-BCP) and subsequently study their structure–property relationships. To achieve this goal, we employed the stereoselective ROP of *rac*-8DL^Pr^ and *meso*-8DL^Pr^ (Pr = *n*-propyl group substituted on the diolide ring, or the side-chain group attached to the PHA main-chain) and chemoselective copolymerization of *rac*-8DL^Pr^ with *rac*-8DL^Me^, using discrete [Y] precatalysts^48–52^ (Figure 1).

**Figure 1:**
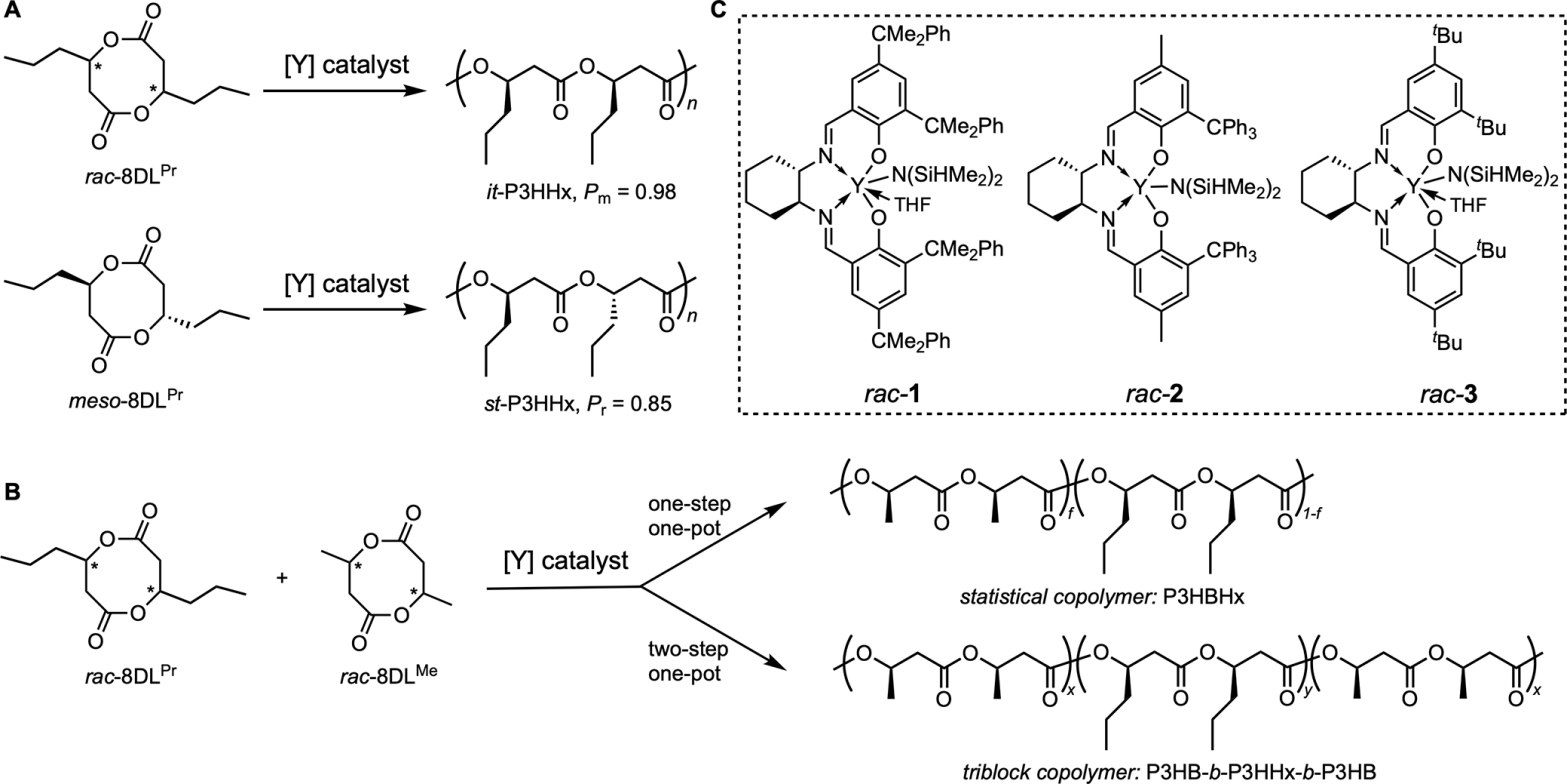
Outlined chemocatalytic route to 3HHx-based PHAs. (A) Stereoselective ROP of *rac*-8DL^Pr^ and *meso*-8DL^Pr^ by [Y] catalysts to produce *it*-P3HHx and *st*-P3HHx, respectively. (B) Stereoselective copolymerization of *rac*-8DL^Pr^ with *rac*-8DL^Me^ to isotactic statistical copolymer P3HBHx and tri-BCP P3HB-*b*-P3HHx-*b*-P3HB via two different monomer addition protocols. (C) Chemical structures of [Y] racemic precatalysts employed in this study.

## Materials and Methods

### Materials

All syntheses and manipulations of air- and moisture-sensitive chemicals and materials were carried out in flamed Schlenk-type glassware on a dual-manifold Schlenk line or in an inert gas (Ar or N_2_)-filled glovebox. HPLC-grade organic solvents were first sparged extensively with nitrogen during filling 20 L solvent reservoirs and then dried by passage through activated alumina (for CH_2_Cl_2_), followed by passage through Q-5 supported copper catalyst (for toluene and hexanes) stainless steel columns. Benzene-*d*_6_ was dried over sodium/potassium alloy and filtered, whereas CD_2_Cl_2_ and CDCl_3_ were dried over CaH_2_, vacuum-distilled, and stored over activated Davison 4 Å molecular sieves.

Yttrium chloride YCl_3_ and lanthanum chloride LaCl_3_ were purchased from Sigma–Aldrich Chemical Co., and used as received. Benzyl alcohol was purchased from Alfa Aesar Chemical Co., purified by distillation over CaH_2_, and stored over activated Davison 4 Å molecular sieves. Dimethyl succinate, sodium methoxide, and 3-chloroperoxybenzoic acid (*m*CPBA, 70–75%) were purchased from Fisher Scientific Co., and used as received. Dimethyl 2,5-dioxocyclohexane-1,4-dicarboxylate was purchased from TCI Chemicals and used as received. Propyl iodide was purchased from Acros Organics and used as received. Literature procedures^39,54^ were employed for the preparation of *rac*-8DL^Me^ and modified for the synthesis of new *rac*-8DL^Pr^. The yttrium and lanthanum complexes were prepared according to their respective literature procedures: Y­[N­(SiHMe_2_)_2_]_3_(THF)_2_, La­[N­(SiHMe_2_)_2_]_3_(THF)_2_,^55^ and complexes **1**–**3**.^56,57^

### General Polymerization Procedures

Polymerizations were performed in 5.5 mL glass reactors inside the inert glovebox at RT (∼23 °C). The reactor was charged with a predetermined amount of catalyst or initiator and solvent (as specified in the polymerization tables) in a glovebox. The mixture was stirred at RT for 10 min, and polymerization was initiated by rapid addition to an 8DL monomer. After a desired time period, the polymerization was immediately quenched by addition of 0.5 mL of benzoic acid/chloroform (10 mg mL^–1^) and a 0.02 mL of aliquot was taken from the reaction mixture and prepared for ^1^H NMR analysis to obtain the percent monomer conversion data. The quenched mixture was then precipitated into 50 mL of cold methanol while being stirred, filtered, washed with cold methanol to remove any unreacted monomer, and dried in a vacuum oven at RT overnight to a constant weight.

### Absolute Molecular Weight Measurements

Measurements of polymer absolute weight-average molecular weight (*M*_w_), number-average molecular weight (*M*_n_), and dispersity indices (*D̵* = *M*_w_/*M*_n_) were performed via size exclusion chromatography (SEC). The SEC instrument consisted of an Agilent HPLC system equipped with one guard column and three PLgel 5 μm mixed-C gel permeation columns and coupled with a Wyatt DAWN HELEOS II multi (18)-angle light scattering detector and a Wyatt Optilab TrEX dRI detector; the analysis was performed at 40 °C using chloroform as the eluent at a flow rate of 1.0 mL min^–1^, using Wyatt ASTRA 7.1.3 molecular weight characterization software. Polymer solutions were prepared in chloroform and injected into the dRI detector by Harvard Apparatus pump 11 at a flow rate of 0.30 mL min^–1^.

### Spectroscopic Characterizations

NMR spectra were recorded on a Bruker AV-III 400 MHz spectrometer (400 MHz, ^1^H; 100 MHz, ^13^C). Chemical shifts for ^1^H and ^13^C spectra were referenced to internal solvent resonances and are reported as parts per million relative to that of SiMe_4_. The [*rr*] (the syndiotactic triad made up of two adjacent *rac* diads probability of *rac* linkages between 3HH units) value of P3HH was calculated according to the integration area of *rr*, *mr,* and *rm* triads [A­([*rr*]), A­([*mr*]), A­([*rm*])] of the carbonyl group region at δ169.1 ppm, that is [*rr*] = A­([*rr*])/[A­([*rr*]), A­([*mr*]), A­([*rm*])]. DOSY NMR spectra were recorded on a Bruker AV-III 400 MHz spectrometer, and analyses were performed at a steady temperature of 25 °C with at least 16 gradient increments using the Dbppste_cc sequence.

### Thermal Analysis

Melting transition (*T*_m_) and glass transition (*T*_g_) temperatures were measured by differential scanning calorimetry (DSC) on an Auto Q20, TA Instrument. All *T*_m_ and *T*_g_ values were obtained from a second scan after the thermal history was removed from the first scan unless noted otherwise. The second heating rate was 10 °C/min and cooling rate was 10 °C/min unless indicated otherwise in the polymerization tables. This heating and cooling rate was used because of the relatively low crystallinity of the resultant polymer and also as a standard condition to compare other chemically synthesized PHAs in our lab. Decomposition temperatures (*T*_d_, defined by the temperature of 5% weight loss) and maximum rate decomposition temperatures (*T*_max_) of the polymers were measured by thermal gravimetric analysis (TGA) on a Q50 TGA Analyzer, TA Instrument. Polymer samples were heated from ambient temperature to 700 °C at a heating rate of 10 °C min^–1^. Values of *T*_max_ were obtained from derivative (wt %/°C) vs temperature (°C) plots, while *T*_d_ and *T*_onset_ values (initial and end temperatures) were obtained from wt % vs temperature (°C) plots.

### Mechanical Analysis

Tensile stress/strain testing was performed by an Instron 5966 universal testing system (10 kN load cell) on dog-bone-shaped test specimens (ASTM D638 standard; Type V) prepared via compression molding using a Carver Bench Top Laboratory Press (Model 4386) equipped with a two-column hydraulic unit (Carver, Model 3912, maximum force 24000 psi). Isolated polymer materials were loaded between nonstick Teflon paper sheets into a stainless-steel mold with inset dimensions 30 × 73.5 × 0.87 mm fabricated in house and compressed between two 6 in. × 6 in. steel electrically heated platens (EHPs) clamp force 3000 psi, at temperature 10 °C higher than each material’s respective *T*_m_. Specimens for analysis were cut using an ASTM D638-5-IMP cutting die (Qualitest) to standard dimensions. Mechanical behavior was averaged for all the specimens measured for each individual species investigated. Thickness (0.34 ± 0.01 mm), width (3.18 mm), and grip length (26.4 ± 0.2 mm) of the measured dog-bone specimens were measured for normalization of data by the Bluehill measurement software (Instron). Test specimens were affixed into the screw-tight grip frame. Tensile stress and strain were measured to the point of material break at a grip extension speed of 5.0 mm min^–1^ under ambient conditions.

## Results and Discussion

### Stereoselective Polymerization of *rac*-8DL^Pr^ and *meso*-8DL^Pr^

The synthesis of new dipropyl-substituted diolide monomer 8DL^Pr^ was accomplished by following previously reported synthetic procedures for the synthesis and separation of *rac*-8DL^R^ and *meso*-8DL^R^, either from biosourced dimethyl succinate or dimethyl 2,5-dioxocyclohexane-1,4-dicarboxylate, both of which are commercially available.^40^ The overall yield for the isolated pure *rac*-8DL^Pr^ and *meso*-8DL^Pr^ was 51% and 36%, respectively, based on the dimethyl dicarboxylate starting material (see the Supporting Information for details).

Yttrium (Y) and lanthanum (La) precatalysts supported by *C*_2_-symmetric *N*,*N′*-*bis*(salicylidene)­cyclohexanediimine (salcy) ligands catalyze the stereoselective ROP of *rac*-8DL^R^, producing highly stereoregular PHAs. The most sterically bulky complex **2** with trityl groups on the *ortho*-phenoxy positions on the salcy ligand exhibits exceptional activity and stereoselectivity for the ROP of *rac*-8DL^Me^ but shows significantly lower activity toward longer alkyl-substituted diolides.^41^ In contrast, the less sterically encumbered complex **1**, with the cumyl-substituted salcy ligand, shows high activity and stereoselectivity toward the bulkier diolide monomers and enables efficient copolymerization of monomers with varying steric bulk. For this study, [Y]-based silylamide precatalysts were activated in situ with 1 equiv of benzyl alcohol (BnOH) to generate the corresponding benzyloxy catalyst, which have been shown to be highly active for the stereoselective polymerization of *rac*-8DL^Me^ in dichloromethane (DCM), affording *it*-P3HB with high *P*_m_ values: 0.96 by *rac*-**1**, >0.99 by *rac*-**2**, and 0.89 by *rac*-**3**). For the ROP of *rac*-8DL^Pr^ in DCM at room temperature (∼23 °C), *rac*-**1** with cumyl substituents on the ligand of moderate sterics mediated rapid polymerization, yielding *it*-P3HHx with near-quantitative isotacticity, *P*_m_ = 0.98 (run 1, Table 1), as determined by ^13^C NMR analysis (Figure 2A). When adjusting the [*rac*-8DL^Pr^]/[**1**] ratio from 200:1 to 2400:1 (i.e., 41.7 ppm catalyst loading), *it*-P3HHx with a high molar mass was obtained, with *M*_n_ = 501 kg/mol and *D̵* = 1.31 in multigram scale (run 2, Table 1). On the other hand, the most sterically hindered complex *rac*-**2** of the catalyst series is completely inactive, even after an extended time period of 24 h (run 3, Table 1), highlighting the importance of steric matching between the catalyst structure and the currently bulky monomer structure of *rac*-8DL^Pr^ (relative to *rac*-8DL^Me^). For the ROP of *meso*-8DL^Pr^ in DCM at room temperature, *rac*-**1** also mediated rapid polymerization (100% monomer conversion in 30 min), yielding syndiotactic *st*-P3HHx (*M*_n_ = 38.1 kg/mol, *D̵* = 1.29) with a syndiotacticity of *P*_r_ (the probability for *racemic* (*RS* or *SR*) placement of the two adjacent monomer repeating units) = 0.85 (Figures 2B and S4), while *rac*-**2** again is unable to initiate the ROP of *meso*-8DL^Pr^ (runs 4–5, Table 1).

**Table 1:** Selected Results for ROP of *rac*-8DL^Pr^ and *meso*-8DL^Pr^ under Different Conditions^a^

run	cat	monomer (M)	[M]/[Cat]	time (h)	conv. (%)^b^	*M*_n_ ^c^ (kg/mol)	*D̵* (*M*_w_/*M*_n_)^c^	*T*_*g*_ ^d^ (°C)	*T*_m_ ^d^ (°C)	*P*_m_ (*P*_r_)^e^
1	**1**	*rac*-8DL^Pr^	200/1	0.5	100	60.5	1.03	–18	-	0.98
2	**1**	*rac*-8DL^Pr^	2400/1	0.5	100	501	1.31	–18	-	0.98
3	**2**	*rac*-8DL^Pr^	200/1	24	0	-	-	-	-	-
4	**1**	*meso*-8DL^Pr^	200/1	0.5	100	38.1	1.29	–14	66	(0.85)
5	**2**	*meso*-8DL^Pr^	200/1	24	0	-	-	-	-	-

a Conditions: monomer (M) = 0.5 mmol, *V*_solvent_ = 0.5 mL, DCM (CH_2_Cl_2_) as the solvent, BnOH (1 equiv relative to the precatalyst complex) as the initiator, ambient temperature (∼23 °C).

b Monomer conversion measured by ^1^H NMR spectra of the quenched solution in benzoic acid/chloroform.

c *M*_w_, *M*_n_, and *D̵* values determined by size-exclusion chromatography (SEC) coupled with an 18-angle light scattering detector at 40 °C in chloroform.

d Measured by differential scanning calorimetry (DSC) with a cooling and heating rate of 10 °C/min.

e Determined by ^13^C NMR spectrum (CDCl_3_, 23 °C).

**Figure 2:**
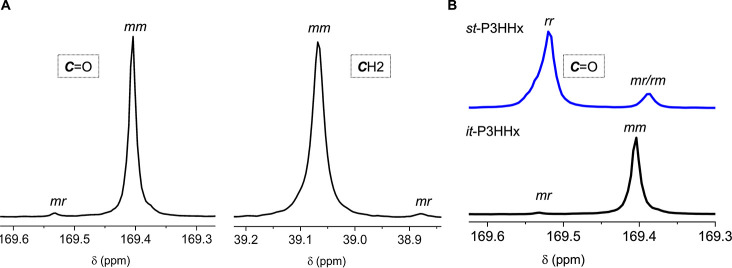
(A) Representative ^13^C NMR spectra (CDCl_3_) of *it*-P3HHx in the regions of tacticity-sensitive carbonyl and methylene groups, produced by [*rac*-8DL^Pr^]/[**1**] = 200/1 in DCM at RT (run 1, Table 1). (B) Overlay of ^13^C NMR spectra (CDCl_3_) of *it*-P3HHx and *st*-P3HHx in the carbonyl region.

### Copolymerization of *rac*-8DL^Me^ and *rac*-8DL^Pr^

To modulate the material properties of *it*-P3HHx, which is an amorphous polymer, *rac*-8DL^Me^ and *rac*-8DL^Pr^ were copolymerized in a one-step, one-pot reaction in a 19/1 feed ratio using 0.5 mol % catalyst loading. The reaction reached complete monomer conversion in 12 min (run 1, Table 2). The resulting copolymer P3HBHx_6.2%_ (6.2% denotes the incorporated molar ratio of *rac*-DL^Pr^, i.e., mol % of 3HHx units incorporated) had a *M*_n_ of 65.0 kg/mol and *D̵* of 1.10. Consistent with our previous observations on copolymerization of *rac*-8DL^Me^ with other alkyl-substituted diolides,^41^ the resulting PHA copolymer P3HBHx is a statistical copolymer. This characterization was further supported by the presence of distinct 3HB-3HHx and 3HHx-3HB signals in the ^13^C NMR spectrum (Figure 3A). With a higher monomer/catalyst/initiator ratio of 2400/2/1, the resulting copolymer P3HBHx_5.9%_ reached a high *M*_n_ of 551 kg/mol and *D̵* of 1.29 (run 2, Table 2). To make the copolymer more flexible, we gradually increased the incorporation ratio of *rac*-8DL^Pr^ (i.e., 3HHx units). Switching the feed ratio of [*rac*-8DL^Me^]/[*rac*-8DL^Pr^] to 9/1, both monomers achieved full conversion in 30 min (runs 3–4, Table 2), producing copolymer P3HBHx_9.2%_ with *M*_n_ = 444 kg/mol and *D̵* = 1.19. The copolymer P3HBHx_19%_ with *M*_n_ = 25.0 kg/mol and *D̵* = 1.02 was obtained when a 4/1 feed ratio of [*rac*-8DL^Me^]/[*rac*-8DL^Pr^] was used (run 5, Table 2). When the [*rac*-8DL^Me^]/[*rac*-8DL^Pr^] feed ratio was adjusted to 1/1, near-quantitative monomer conversion can also be achieved, affording P3HBHx_48%_ with *M*_n_ = 23.1 kg/mol and *D̵* = 1.39 (run 6, Table 2).

**Table 2:** Selected Results for Copolymerization of *rac*-8DL^Me^ with *rac*-8DL^Pr^ under Different Conditions^a^

run	cat	[*rac*-8DL^Me^ + *rac*-8DL^Pr^]/[Cat]/[I]	*rac*-8DL^Me^/*rac*-8DL^Pr^	time (h)	conv. (%)^b^	3HHx Content^c^ (%)	*M*_n_ ^d^ (kg/mol)	*D̵* (*M*_w_/*M*_n_)^d^	*T*_g_ ^e^ (°C)	*T*_m_ ^e^ (°C)	Δ*H*_f_ ^e^ (J/g)
1	**1**	200/1/1	19/1	0.2	100	6.2	65.0	1.10	3.1	147	45.6
2^f^	**1**	2400/2/1	19/1	0.2	100	5.9	551	1.29	2.7	148	40.0
3	**1**	200/1/1	9/1	0.5	100	9.5	27.9	1.08	0.2	141	48.7
4^f^	**1**	2400/2/1	9/1	0.5	100	9.2	444	1.19	1.2	140	38.1
5	**1**	200/1/1	4/1	24	100	19.0	25.0	1.02	–2.6	124	20.6
6	**1**	200/1/1	1/1	48	>98	48.0	23.1	1.39	–7.2	72.3	21.7
7^g^	**1**	1200/2/1	7/3	24	100	28.9	163	1.23	–19/5.5	154	28.3
8^g^	**1**	1200/2/1	3/7	6	100	69.8	168	1.29	–18	154	15.2

a Conditions: *rac*-8DL^Me^ + *rac*-8DL^Pr^ = 0.5 mmol, *V*_solvent_ = 0.5 mL, CH_2_Cl_2_ as the solvent, BnOH as the initiator, precatalyst **1** and initiator amount varied according to the [monomer]/[Cat]/[BnOH] ratio, ambient temperature (∼23 °C).

b Monomer conversion measured by ^1^H NMR spectra of the quenched solution in benzoic acid/chloroform.

c 3HHx content measured by ^1^H NMR of the isolated copolymer.

d *M*_w_, *M*_n_, and *D̵* values determined by SEC coupled with an 18-angle light scattering detector at 40 °C in chloroform.

e Measured by DSC with the cooling and heating rate of 10 °C/min.

f 6 mmol (1.1 g of total monomer) scale.

g BDM was used as an initiator to synthesize discrete tri-BCP.

**Figure 3:**
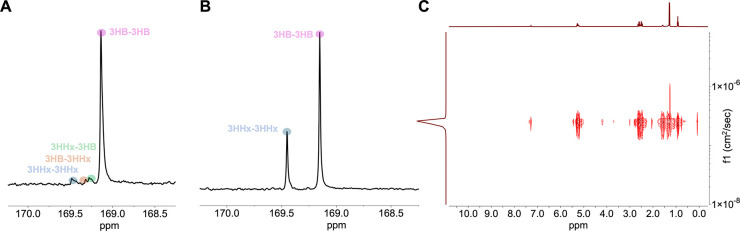
(A) The expanded carbonyl region of the ^13^C NMR spectrum (CDCl_3_) of statistical copolymer P3HBHx_9.2%_ (run 4, Table 2). (B) The expanded carbonyl region of the ^13^C NMR spectrum (CDCl_3_) of tri-BCP P3HB-*b*-P3HHx-*b*-P3HB (28.9% 3HHx incorporation, run 7, Table 2). (C) DOSY NMR spectrum (CDCl_3_, 23 °C) of P3HB-*b*-P3HHx-*b*-P3HB (run 7, Table 2).

The synthesis and characterization of hard–soft–hard ABA tri-BCP as a route to fully PHA-based thermoplastic elastomers were also investigated. Here, we synthesized a discrete ABA tri-BCP architecture, P3HB-*b*-P3HHx-*b*-P3HB, using blocks with targeted comonomer ratios of 7/3 and 3/7 (*rac*-8DL^Pr^/*rac*-8DL^Me^), where *it*-P3HB, with its high crystallinity and *T*_m_, was selected as the hard A block, while the *n*-propyl-substituted amorphous P3HHx was selected as the soft B block due to its low *T*_g_ (−18 °C). Owing to the livingness of the current ROP system, a two-step, one-pot sequential monomer addition procedure enabled the controlled synthesis of tri-BCP architectures. Specifically, using 1,4-benzenedimethanol (BDM) as a difunctional initiator, ABA tri-BCP was synthesized efficiently through this block copolymerization procedure. First, BDM was combined with two equiv of precatalyst **1** to generate the active species in situ, which was then used to polymerize *rac*-8DL^Pr^ to generate the middle soft B-block. Upon complete consumption of *rac*-8DL^Pr^, *rac*-8DL^Me^ was introduced to form the two outer hard A-blocks, completing the construction of the ABA tri-BCP architecture. The carbonyl region of the ^13^C NMR spectrum of the resulting tri-BCP reveals well-resolved block-specific resonances, indicating no transesterification or randomization between the blocks (Figures 3B, S16, and S18). Furthermore, diffusion-ordered spectroscopy (DOSY) experiments revealed a single diffusion coefficient for the triblock copolymers (Figures 3C and S20), in contrast to a physical blend (mixture) of P3HB and P3HHx homopolymers, which displayed two distinct diffusion coefficients (Figure S19). These informative characterization results, together with the observed unimodal molar mass distribution with low *D̵* values (1.23–1.28) for the as-synthesized (without going through solvent fractionation etc.) tri-BCP (Figures S31 and S32), provide conclusive evidence for successful ABA triblock formation.

### Microstructures of 3HHx-Based Copolymers

The microstructures of the P3HBHx copolymers of *rac*-8DL^Me^ with *rac*-8DL^Pr^ were investigated by ^1^H and ^13^C NMR spectroscopy. While the ^1^H NMR spectrum of representative statistical copolymer P3HBHx with a 3HHx incorporation of 9.2% (run 4, Table 2) offers no microstructure information on the copolymer (Figure S9), the ^13^C NMR spectrum provides a detailed insight into the copolymer microstructure (Figure 3A). The expanded carbonyl resonances (169.0–169.6 ppm) were resolved into four different group peaks, arising from different dyad sequences of 3HB and 3HHx units (3HHx-3HHx, 169.4 ppm; 3HB-3HHx, 169.3 ppm; 3HHx-3HB, 169.2 ppm; and 3HB-3HB, 169.1 ppm). The presence of 3HB-3HHx and 3HHx-3HB alternating sequences further confirmed the statistical incorporation of 3HHx in the resulting copolymer P3HBHx_9.2%_. In sharp contrast, the carbonyl region of the ^13^C NMR spectrum for ABA tri-BPC P3HB-*b*-P3HHx-*b*-P3HB shows only two main resonances corresponding to the P3HB and P3HHx blocks, with no dyad splitting, consistent with discrete block formation and the absence of block randomization (Figures 3B and S16).

### Thermal Properties of 3HHx-Based Copolymers

Isotactic homopolymer *it*-P3HHx showed a low *T*_g_ of −18 °C but no *T*_m_ on differential scanning calorimetry (DSC) scans, indicating its amorphous nature that lacks crystallinity, presumably due to the long flexible side *n*-propyl chains that prevent the crystallization. On the other hand, syndiotactic *st*-P3HHx displayed an endotherm peak, giving a *T*_m_ of 66 °C on the first DSC heating scan; however, no *T*_m_ endotherm was observed on the second heating scan, indicating that *st*-P3HHx is a semicrystalline material but with a slow crystallization rate (Figure 4A). The *T*_m_ and *T*_g_ endothermic transitions of statistical copolymers P3HBHx_x%_ are compared and overlaid in Figure 4B. These copolymers show a clear compositional trend, with both *T*_m_ and *T*_g_ decreasing as the 3HHx content increases. For example, P3HBHx_9.5%_ gives a *T*_g_ of 0 °C and a *T*_m_ of 141.2 °C as well as a crystallization temperature (*T*_c_) of 62 °C (enthalpy of fusion (Δ*H*_f_) = 60.4 J/g). Increasing the 3HHx content to 14.7%, P3HBHx_14.7%_ shows a *T*_g_ of −1.6 °C and *T*_m_ of 134.1 °C, comparable to those of commercial P3HBHx.^27^

**Figure 4:**
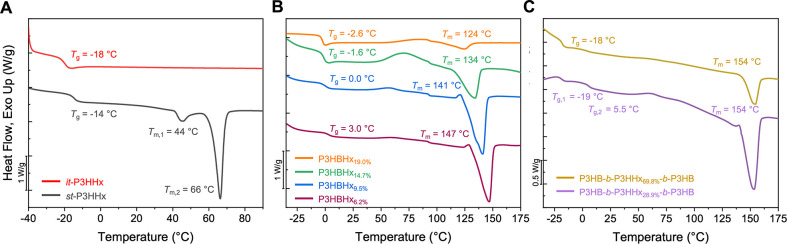
(A) DSC curves of *it*-P3HHx (*P*_m_ = 0.98, Run 1, Table 1) and *st*-P3HHx (*P*_r_ = 0.85, Run 4, Table 1). (B) DSC curves of the copolymers P3HBHx_x%_ (x% indicates the incorporation ratio of 3HHx). (C) DSC curves of tri-BCP P3HB-*b*-P3HHx-*b*-P3HB.

The tri-BCP P3HB-*b*-P3HHx-*b*-P3HB with 29% 3HHx incorporation displayed two *T*_g_ endotherms: −19 °C for the P3HHx block and 5 °C for the P3HB block. The *T*_c_ is 64 °C and *T*_m_ is 154 °C with Δ*H*_f_ = 28.3 J g^–1^ (Figure 4C). The tri-BCP with 70% P3HHx midblock showed a single apparent *T*_g_ at −18 °C corresponding to the P3HHx block, along with a *T*_c_ of 57 °C and the same *T*_m_ but lower crystallinity (154 °C, Δ*H*_f_ = 15.2 J g^–1^), due to the increased 3HHx content (Figure 4C). Overall, the combined low *T*_g_ (−18 °C) and high *T*_m_ (154 °C) of the tri-BCP PHA further expands its application temperature window.

Thermogravimetric analysis (TGA) of *st*-P3HHx (*M*_n_ = 38.1 kg/mol, *D̵* = 1.29, *P*_r_ = 0.85) showed a degradation temperature at 5% mass loss (*T*_d,5%_) of 248 °C and a maximum degradation temperature (*T*_max_) of 271 °C (Figure 5A). In comparison, the TGA of *it*-P3HHx (*M*_n_ = 501 kg/mol, *D̵* = 1.31, *P*_m_ = 0.98) gave a 24 °C lower *T*_d_ of 224 °C and a 9 °C lower *T*_max_ of 262 °C (Figure S21), indicating noticeable effects of tacticity on the P3HHx thermal degradation behavior. Moreover, the statistical copolymer P3HBHx_9.2%_ (*M*_n_ = 444 kg/mol, *D̵* = 1.19) also displayed only one degradation step with a *T*_d_ of 242 °C and a *T*_max_ of 262 °C, further confirming its statistical structure (Figure 5B).

**Figure 5:**
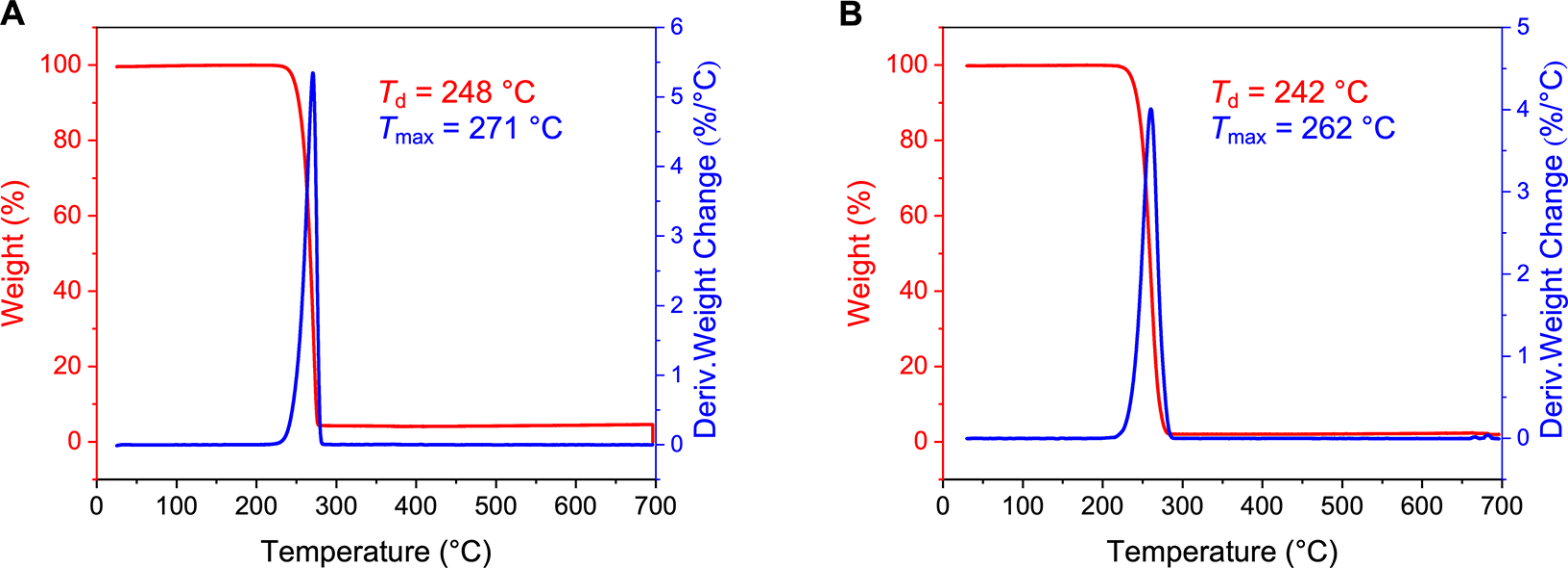
TGA curves: (A) *st*-P3HHx (*M*_n_ = 38.1 kg/mol, *D̵* = 1.29, *P*_r_ = 0.85) and (B) statistical copolymer P3HBHx_9.2%_ (*M*_n_ = 444 kg/mol, *D̵* = 1.19).

### Mechanical Properties of 3HHx-Based Copolymers

The statistical copolymer P3HBHx_5.9%_ (*M*_n_ = 551 kg/mol, *D̵* = 1.29), synthesized by copolymerization of *rac*-8DL^Me^ with *rac*-8DL^Pr^ on a multigram scale and processed into ASTM dog-bone-shaped specimens by compression molding, is stiff with a high Young’s Modulus (*E* = 1390 ± 127 MPa) and strong with a yield stress (σ_*y*_) of 23.2 ± 0.5 MPa, but it is brittle with a small elongation at break (ε_B_) of 36.3 ± 13% (Figure 6A). Increasing the 3HHx incorporation to 9.2%, P3HBHx_9.2%_ lowered the modulus (*E* = 949 ± 19 MPa) but gained ultimate tensile strength (σ_B_ = 15 ± 2.6 MPa) and substantially fracture strain (ε_B_ = 315 ± 47%), thus enhancing the overall toughness of the PHA by ∼8 fold (Tables S1 and S2). Notably, with increasing 3HHx incorporation, copolymer P3HBHx_14.7%_ showed an even lower Young’s modulus as expected (*E* = 497 ± 8.2 MPa) but a further enhanced elongation at break (ε_B_ = 445 ± 60%, Table S3). These results show that the mechanical properties of P3HBHx can be largely tuned by modulating the 3HHx incorporation via the copolymerization using different comonomer feed ratios. In addition, P3HBHx can combine the advantages of high Young’s modulus and strength of *it*-P3HB with the high extensibility of P3HHx, giving a PHA that is both strong and tough. For comparison, the copolymer sample P3HBHx_15.2%_ synthesized by copolymerization of *meso*-8DL^Me^ with *rac*-8DL^Pr^ (incorporation of 3HHx = 15.2%, *M*_n_ = 390.1 kg mol^–1^, *D̵* = 1.23) is a soft but tough thermoplastic elastomer with σ_B_ = 24.1 ± 1.80 MPa, *E* = 29.9 ± 5.24 MPa, and ε_B_ = 726 ± 43.3% (Figures 6B and S33, Table S4).

**Figure 6:**
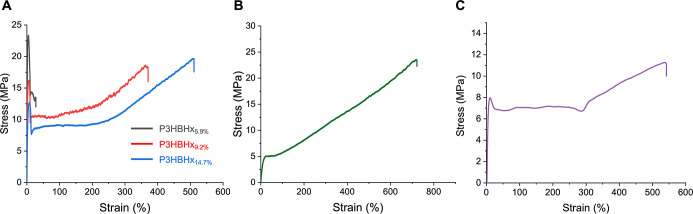
Representative stress–strain curves (5 mm/min, RT) of 3HHx-based PHAs. (A) Statistical copolymer P3HBHx_x%_: P3HBHx_5.9%_, *M*_n_ = 551 kg/mol, *D̵* = 1.29; P3HBH_9.2%_, *M*_n_ = 444 kg/mol, *D̵* = 1.19; P3HBHx_14.7%_, *M*_n_ = 368 kg/mol, *D̵* = 1.13. (B) Statistical copolymer P3HBHx_15.2%_ synthesized by copolymerization of *meso*-8DL^Me^ with *rac*-8DL^Pr^, *M*_n_ = 390.1 kg/mol, *D̵* = 1.23. (C) Tri-BCP P3HB-*b*-P3HHx-*b*-P3HB (28.9% 3HHx incorporation), *M*_n_ = 163 kg/mol, *D̵* = 1.23.

For tri-BCP materials, the P3HB-*b*-P3HHx-*b*-P3HB with a 28.9% P3HHx midblock is a tough thermoplastic with σ_B_ = 10.6 ± 0.5 MPa, *E* = 405 ± 19 MPa, and ε_B_ = 536 ± 76% (Figures 6C and S34, Table S5). In contrast, the tri-BCP with a 69.8% P3HHx midblock is a weak thermoplastic with σ_B_ = 0.60 ± 0.01 MPa, *E* = 5.4 ± 0.2 MPa, and ε_B_ = 486 ± 17% (Figure S35 and Table S6).

## Conclusion

This work presents a chemocatalytic route to commercially implemented and biologically produced P3HHx-based PHA materials. As little is known in the open literature about the structure and property characterizations of discrete, synthetically authenticated homopolymer P3HHx and its copolymers with P3HB, the results reported herein should be of interest to the broad biological and synthetic PHA communities.

Through catalyzed stereoselective ROP of *rac*-8DL^Pr^ and *meso*-8DL^Me^ with chiral [Y]-based catalysts, isotactic and syndiotactic P3HHx homopolymers, *it*-P3HHx and *st*-P3HHx, have been synthesized and comprehensively characterized. While *it*-P3HHx is a low *T*_g_ (−18 °C), amorphous material, *st*-P3HHx is a semicrystalline PHA (*T*_m_ = 66 °C), despite its low crystallization rate. By stereoselective copolymerization of *rac*-8DL^Pr^ with *rac*-8DL^Me^ with different monomer feed ratios, a series of isotactic statistical copolymers, P3HBHx, with high molar mass (*M*_n_ up to 551 kDa) and controllable levels of 3HHx incorporation (derived from the ROP of *rac*-8DL^Pr^) has been synthesized and extensively characterized. The thermal and mechanical properties of P3HBHx can be largely tuned by the percentage of the 3HHx incorporation: P3HBHx_5.9%_ (*M*_n_ = 551 kg/mol) is stiff, strong, and rigid (*E* = 1.39 GPa, σ_*y*_ = 23.2 MPa, ε_B_ = 36%), P3HBHx_9.2%_ (*M*_n_ = 444 kg/mol) is less stiff and much more flexible (*E* = 949 MPa, ε_B_ = 315%), and P3HBHx_14.7%_ (*M*_n_ = 368 kg/mol) is the softest and most flexible of the series (*E* = 497 MPa, ε_B_ = 445%).

Notably, P3HBHx_9.2%_ is a strong and tough thermoplastic with an σ_B_ of 18.6 MPa, which combines the advantages of the high modulus of *it*-P3HB and the ductility of P3HHx, coupled with a near-zero *T*_g_ and a high *T*_m_ (140 °C); thus, it could find potential applications in packaging where biodegradability and stability at high temperatures are of importance. Further expansion of application temperature windows is realized by the synthesis of discrete hard–soft–hard tri-BCP, P3HB-*b*-P3HHx-*b*-P3HB, currently only accessible via the chemocatalytic route presented herein. The combination of the lower *T*_g_ (−18 °C) and higher *T*_m_ (154 °C) of tri-BCP PHA offers unique (both low- and high-temperature stability) properties for this architecture. Overall, these findings highlight the stereomicrostructural and architectural versatility of chemocatalytic routes to PHA homopolymers and copolymers, and in turn they can be effectively utilized to largely tune the PHA thermal properties and mechanical performance.

## Supplementary Material



## Notes

Detailed procedures for monomer synthesis, characterizations of synthesized monomers and polymers, and additional supporting figures and tables (PDF)
