# Supplementary material for: A Chemocatalytic Route to Stereoregular Poly(3-hydroxyhexanoate) and Its Statistical and Tri-Block Copolymers
Source: Biomacromolecules. 2026 Feb 23;27(3):2397–405. doi: 10.1021/acs.biomac.6c00167 (PMC12977045; doi:10.1021/acs.biomac.6c00167)
Supplement: Supplementary file 1 [file bm6c00167_si_001.pdf]

Supporting Information

A Chemocatalytic Route to Stereoregular Poly(3-hydroxyhexanoate) and Its Statistical and Tri-Block Copolymers

Min Zhu,† Maëlle T. Gace,† Zhen Zhang,† Eugene Y.-X. Chen*

Department of Chemistry, Colorado State University, Fort Collins, CO 80523-1872, United States

Corresponding Author; email: eugene.chen@colostate.edu

Table of Contents

Synthesis of *rac*-8DL^{Pr} and *meso*-8DL^{Pr}....................4

Figure S1. ^{1}H NMR ($CDCl_3$, 23 °C) of *meso*-8DL^{Pr}. 6

Figure S2. ^{1}H NMR ($CDCl_3$, 23 °C) of *rac*-8DL^{Pr}. 6

Figure S3. ^{1}H NMR ($CDCl_3$, 23 °C) of *st*-P3HHx....................7

Figure S4. ^{13}C NMR ($CDCl_3$, 23 °C) of *st*-P3HHx....................7

Figure S5. ^{1}H NMR ($CDCl_3$, 23 °C) of *it*-P3HHx. 8

Figure S6. ^{13}C NMR ($CDCl_3$, 23 °C) of *it*-P3HHx. 8

Figure S7. ^{1}H NMR ($CDCl_3$, 23 °C) of statistical copolymer P3HBHx (5.9% 3HHx incorporation)..........9

Figure S8. ^{13}C NMR ($CDCl_3$, 23 °C) of statistical copolymer P3HBHx (5.9% 3HHx incorporation).........9

Figure S9. ^{1}H NMR ($CDCl_3$, 23 °C) of statistical copolymer P3HBHx (9.2% 3HHx incorporation)........10

Figure S10. ^{13}C NMR ($CDCl_3$, 23 °C) of statistical copolymer P3HBHx (9.2% 3HHx incorporation).....10

Figure S11. ^{1}H NMR ($CDCl_3$, 23 °C) of statistical copolymer P3HBHx (14.7% 3HHx incorporation)....11

Figure S12. ^{13}C NMR ($CDCl_3$, 23 °C) of statistical copolymer P3HBHx (14.7% 3HHx incorporation)...11

Figure S13. ^{1}H NMR ($CDCl_3$, 23 °C) of statistical copolymer P3HBHx (19% 3HHx incorporation).......12

Figure S14. ^{13}C NMR ($CDCl_3$, 23 °C) of statistical copolymer P3HBHx (19% 3HHx incorporation)......12

Figure S15. ^{1}H NMR ($CDCl_3$, 23 °C) of triblock copolymer P3HB-*b*-P3HHx-*b*-P3HB (28.9% 3HHx midblock incorporation). 13

Figure S16. ^{13}C NMR ($CDCl_3$, 23 °C) of triblock copolymer P3HB-*b*-P3HHx-*b*-P3HB (28.9% 3HHx midblock incorporation). 13

Figure S17. ^{1}H NMR ($CDCl_3$, 23 °C) of triblock copolymer P3HB-*b*-P3HHx-*b*-P3HB (69.8% 3HHx midblock incorporation). 14

Figure S18. ^{13}C NMR ($CDCl_3$, 23 °C) of triblock copolymer P3HB-*b*-P3HHx-*b*-P3HB (69.8% 3HHx midblock incorporation). 14

Figure S19. DOSY spectrum ($CDCl_3$, 23 °C) of a binary blend (2:1) mixture of P3HHx and P3HB homopolymers. 15

Figure S20. DOSY spectrum ($CDCl_3$, 23 °C) of P3HB-*b*-P3HHx-*b*-P3HB (Run 8, Table 2). 15

Figure S21. TGA curve of homopolymer *it*-P3HBHx (M_n = 501 kg mol^{-1}, $Đ$ = 1.31, P_m = 0.98). 16

Figure S22. TGA curve of statistical copolymer P3HBHx (M_n = 551 kg mol^{-1}, $Đ$ = 1.29, 5.9% incorporation of 3HHx units). 16

Figure S23. TGA curve of statistical copolymer P3HBHx (M_n = 368 kg mol^{-1}, $Đ$ = 1.13, 14.7% incorporation of 3HHx units). 17

Figure S24. TGA curve of triblock copolymer P3HB-*b*-P3HHx-*b*-P3HB (M_n = 163 kg mol^{-1}, $Đ$ = 1.23, 28.9% incorporation of 3HHx units). 17

Figure S25. TGA curve of triblock copolymer P3HB-*b*-P3HHx-*b*-P3HB (M_n = 168 kg mol^{-1}, $Đ$ = 1.28, 69.8% incorporation of 3HHx units). 18

Figure S26. SEC trace of *it*-P3HHx (M_n = 60.5 kg mol^{-1}, $Đ$ = 1.03) (Run 1, Table 1). 18

Figure S27. SEC trace of *st*-P3HHx (M_n = 38.1 kg mol^{-1}, $Đ$ = 1.29) (Run 4, Table 1). 19

Figure S28. SEC trace of statistical copolymer P3HBHx (M_n = 65 kg mol^{-1}, $Đ$ = 1.10, 6.2% incorporation of 3HHx units) (Run 1, Table 2). 19

Figure S29. SEC trace of statistical copolymer P3HBHx (M_n = 27.9 kg mol^{-1}, $Đ$ = 1.08, 9.5% incorporation of 3HHx units) (Run 3, Table 2). 20

Figure S30. SEC trace of statistical copolymer P3HBHx (M_n = 25.0 kg mol^{-1}, $Đ$ = 1.02, 19% incorporation of 3HHx units) (Run 5, Table 2). 20

Figure S31. SEC trace of triblock copolymer P3HB-*b*-P3HHx-*b*-P3HB (M_n = 163 kg mol^{-1}, $Đ$ = 1.23, 28.9% incorporation of 3HHx units) (Run 7, Table 2). 21

Figure S32. SEC trace of triblock copolymer P3HB-*b*-P3HHx-*b*-P3HB (M_n = 168 kg mol^{-1}, $Đ$ = 1.28, 69.8% incorporation of 3HHx units) (Run 8, Table 2). 21

Figure S33. Stress-strain curves of statistical copolymer P3HBHx copolymerized by *meso*-8DL^{Me} and *rac*-8DL^{Pr} (incorporation of 3HHx = 15.2%, M_n = 390.1 kg mol^{-1}, $Đ$ = 1.23). 22

Figure S34. Stress-strain curves of triblock copolymer P3HB-*b*-P3HHx-*b*-P3HB (28.9% incorporation of 3HHx units) (Run 7, Table 2). 22

Figure S35. Stress-strain curves of triblock copolymer P3HB-*b*-P3HHx-*b*-P3HB (69.8% incorporation of 3HHx units) (Run 8, Table 2). 23

Table S1. Measured tensile behavior of P3HBHx copolymerized by *rac*-$8DL^{Me}$ and *rac*-$8DL^{Pr}$ (3HHx incorporation = 5.9%, M_n = 551 kg mol^{-1}, $Đ$ = 1.29) dog-bone shaped specimens (ASTM D638-5).........24

Table S2. Measured tensile behavior of P3HBHx copolymerized by *rac*-$8DL^{Me}$ and *rac*-$8DL^{Pr}$ (3HHx incorporation = 9.2%, M_n = 444 kg mol^{-1}, $Đ$ = 1.19) dog-bone shaped specimens (ASTM D638-5).........24

Table S3. Measured tensile behavior of P3HBHx copolymerized by *rac*-$8DL^{Me}$ and *rac*-$8DL^{Pr}$ (3HHx incorporation = 14.7%, M_n = 368 kg mol^{-1}, $Đ$ = 1.13) dog-bone shaped specimens (ASTM D638-5).......24

Table S4. Measured tensile behavior of P3HBHx copolymerized by *meso*-$8DL^{Me}$ and *rac*-$8DL^{Pr}$ (3HHx incorporation = 15.2%, M_n = 390.1 kg mol^{-1}, $Đ$ = 1.23) dog-bone shaped specimens (ASTM D638-5)....25

Table S5. Measured tensile behavior of triblock copolymer P3HB-*b*-P3HHx-*b*-P3HB copolymerized by *rac*-$8DL^{Me}$ and *rac*-$8DL^{Pr}$ (3HHx incorporation = 28.9%, M_n = 163 kg mol^{-1}, $Đ$ = 1.23) dog-bone shaped specimens (ASTM D638-5)..........25

Table S6. Measured tensile behavior of triblock copolymer P3HB-*b*-P3HHx-*b*-P3HB copolymerized by *rac*-$8DL^{Me}$ and *rac*-$8DL^{Pr}$ (3HHx incorporation = 69.8%, M_n = 168 kg mol^{-1}, $Đ$ = 1.29) dog-bone shaped specimens (ASTM D638-5)..........25

Synthesis of *rac*-8DL^{Pr} and *meso*-8DL^{Pr}

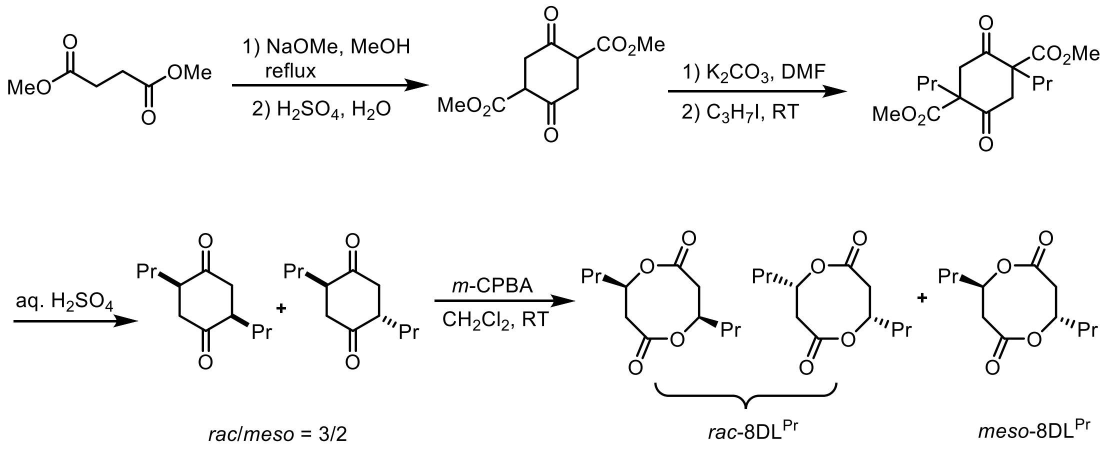

Dimethyl 1,4-dipropyl-2,5-dioxocyclohexane-1,4-dicarboxylate. To a stirred suspension of K_2CO_3 (498 g, 3.6 mol) in 2.5 L DMF under N_2 was added dimethyl 2,5-dioxocyclohexane-1,4-dicarboxylate (274 g, 1.2 mol). After 15 min stirring at room temperature, propyl iodide (468 mL, 4.8 mol) was added dropwise. After 24 h, the mixture was concentrated in vacuo, dissolved in 1200 mL of H_2O, and extracted with CH_2Cl_2 (500 mL x 3). The combined organic layers were washed twice with 10% $Na_2S_2O_3$ solution, washed once with saturated NaCl, dried with anhydrous Na_2SO_4, and evaporated. The solid was washed with hexanes to give 344 g (92%) of the title compound which was directly used for next step without further purification.

***Trans*-2,5-dipropylcyclohexane-1,4-dione.** To a stirred suspension of dimethyl 1,4-dipropyl-2,5-dioxocyclohexane-1,4-dicarboxylate (344 g, 1.1 mol) in 30 mL methanol and 450 g crushed ice, 300 mL of concentrated H_2SO_4 was added. After 15 min of stirring at room temperature, the mixture was heated to 90 °C for 60 h. The acidic solution was cooled to room temperature, neutralized with aq. NaOH (pH 6-7), and extracted with CH_2Cl_2 (350 mL x 3). The combined organic layers were washed twice with saturated NaCl, dried with anhydrous Na_2SO_4, and evaporated. The residue was purified by recrystallization in EtOAc/hexanes to afford the crude compound as a 2:1 mixture of diastereomers which was directly used for next step without further purification.

***Trans*-4,8-dipropyl-1,5-dioxocane-2,6-dione (*meso*-8DL^{Pr}).** To a solution of the *trans*-2,5- dipropyl cyclohexane-1,4-dione (10 g, 51 mmol) in 500 mL of CH_2Cl_2 was added *m*-CPBA (35.2 g, 75%, 153 mmol) in one portion. The pale-yellow solution was stirred at room temperature in the dark for 48 h. The obtained white suspension was diluted with 200 mL of CH_2Cl_2, washed with saturated $NaHCO_3$ solution (100 mL x 3), which contained 5% $Na_2S_2O_3$, dried with anhydrous Na_2SO_4, and evaporated. After recrystallization of the residue (10.1 g) from hexanes/EtOAc (5/1) and recrystallization from hexane 7.2 g of pure *meso*-8DL^{Pr} was obtained (62% yield). ^{1}H NMR (400 MHz, $CDCl_3$) δ 5.14-5.07 (m, 2H), 2.92 (dd, J = 5.6, 5.6 Hz, 2H),

2.54 (dd, J = 7.6, 7.2 Hz, 2H), 1.84-1.75 (m, 2H), 1.63-1.54 (m, 2H), 1.53-1.33 (m, 4H), 0.96 (t, J = 7.2 Hz, 6H).

***Cis*-4,8-dipropyl-1,5-dioxocane-2,6-dione (*rac*-8DL^{Pr}).** The filtrate from the above purification step of *trans*-2,5-dibenzylcyclohexane-1,4-dione was purified by column chromatography to give the *cis*-dione in ~80% racemic content. To a solution of the *cis*-2,5-dipropylcyclohexane-1,4-dione (10 g, 51 mmol) in 500 mL of CH_2Cl_2 was added *m*-CPBA (46 g, 75%, 204 mmol) in one portion. The yellow solution was stirred at room temperature in the dark for 48 h. The obtained white suspension was diluted with 100 mL of CH_2Cl_2, washed with saturated $NaHCO_3$ solution (50 mL x 3), which contained 5% $Na_2S_2O_3$, dried with anhydrous Na_2SO_4, and evaporated. After recrystallization of the residue from hexanes/EtOAc, 8.4 g of pure *rac*-8DL^{Pr} was obtained (72% yield). ^1H NMR (400 MHz, $CDCl_3$) δ 5.21-5.14 (m, 2H), 2.66 (t, J = 10 Hz, 2H), 2.48 (dd, J = 3.2, 3.6 Hz, 2H), 1.81-1.72 (m, 2H), 1.63-1.55 (m, 2H), 1.51-1.36 (m, 4H), 0.96 (t, J = 7.6 Hz, 6H).

Additional Supporting Figures

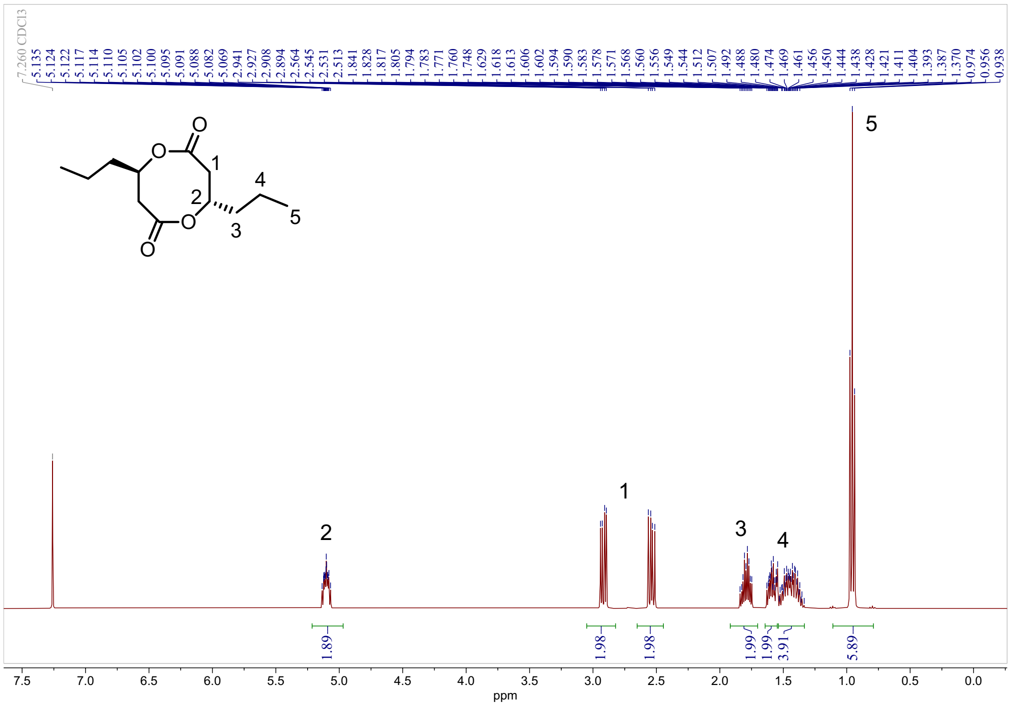

Figure S1. ^{1}H NMR ($CDCl_3$, 23 °C) of *meso*-8DL^{Pr}.

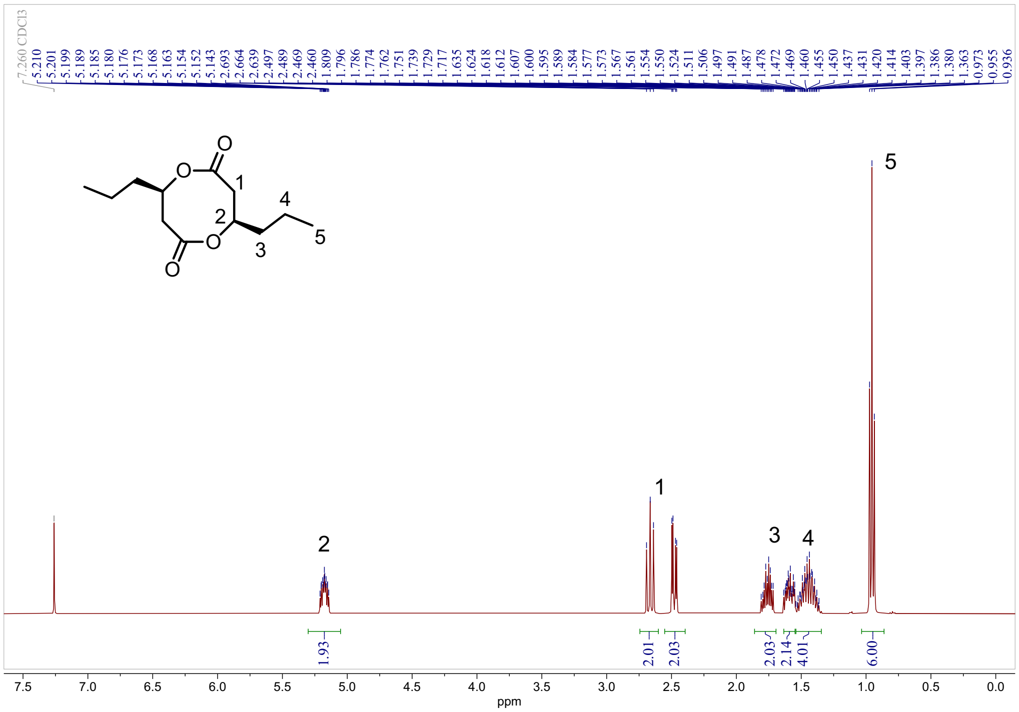

Figure S2. ^{1}H NMR ($CDCl_3$, 23 °C) of *rac*-8DL^{Pr}.

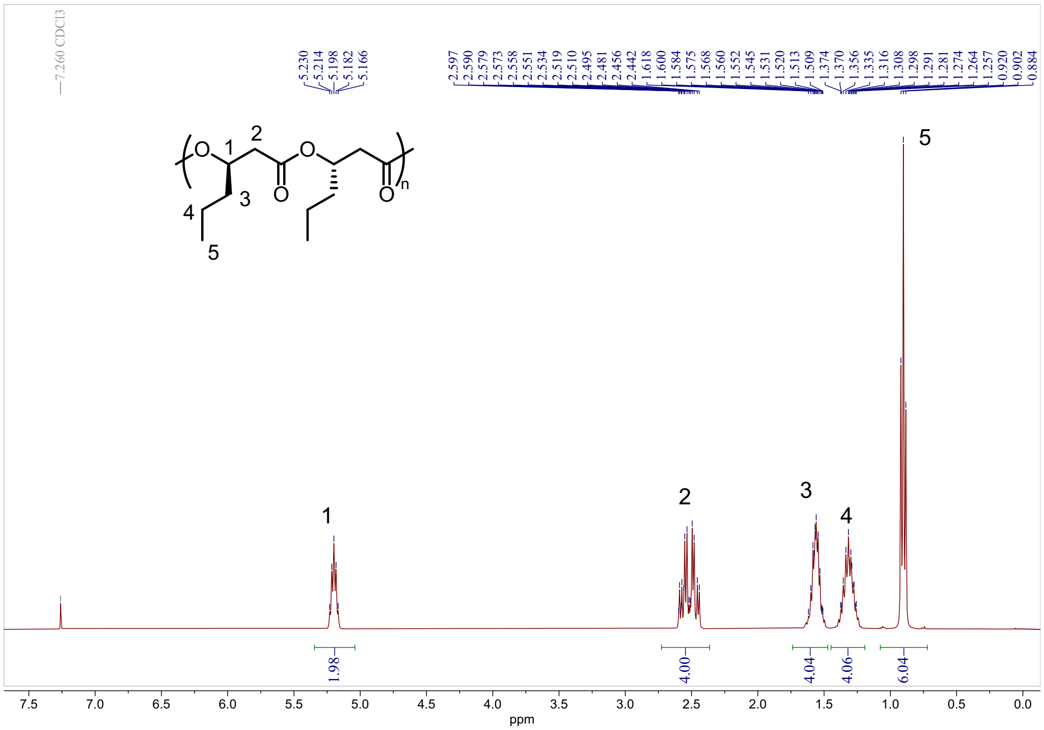

Figure S3. ^{1}H NMR ($CDCl_3$, 23 °C) of *st*-P3HHx.

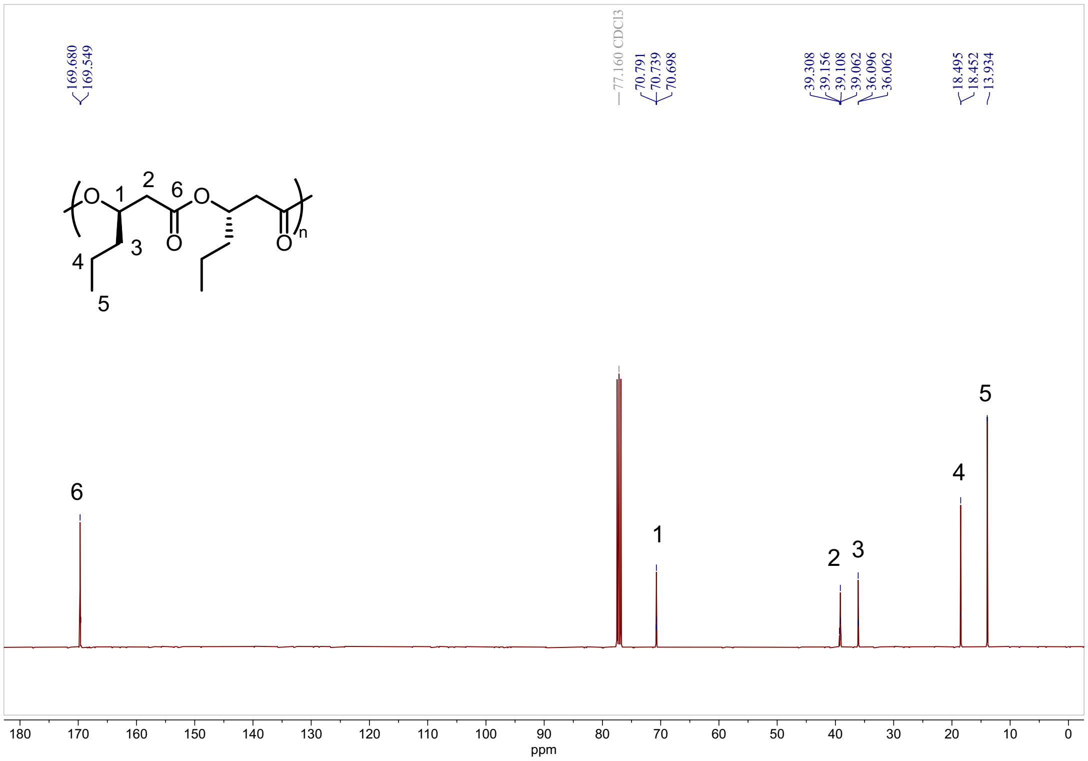

Figure S4. ^{13}C NMR ($CDCl_3$, 23 °C) of *st*-P3HHx.

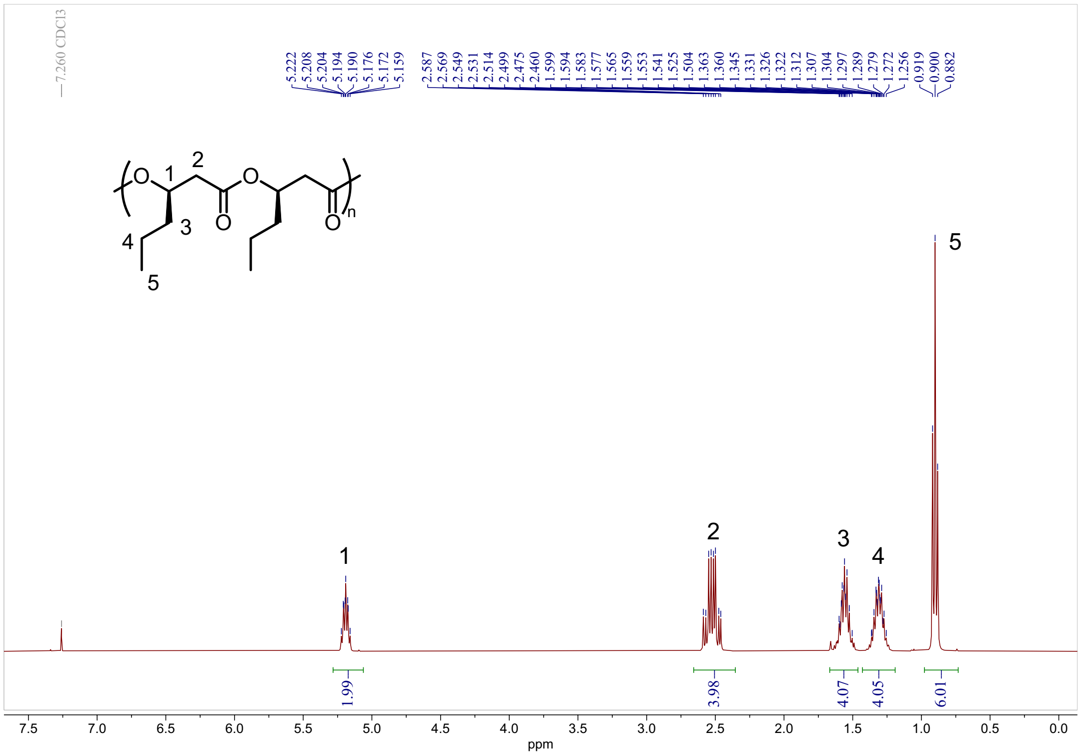

Figure S5. ^{1}H NMR ($CDCl_3$, 23 °C) of *it*-P3HHx.

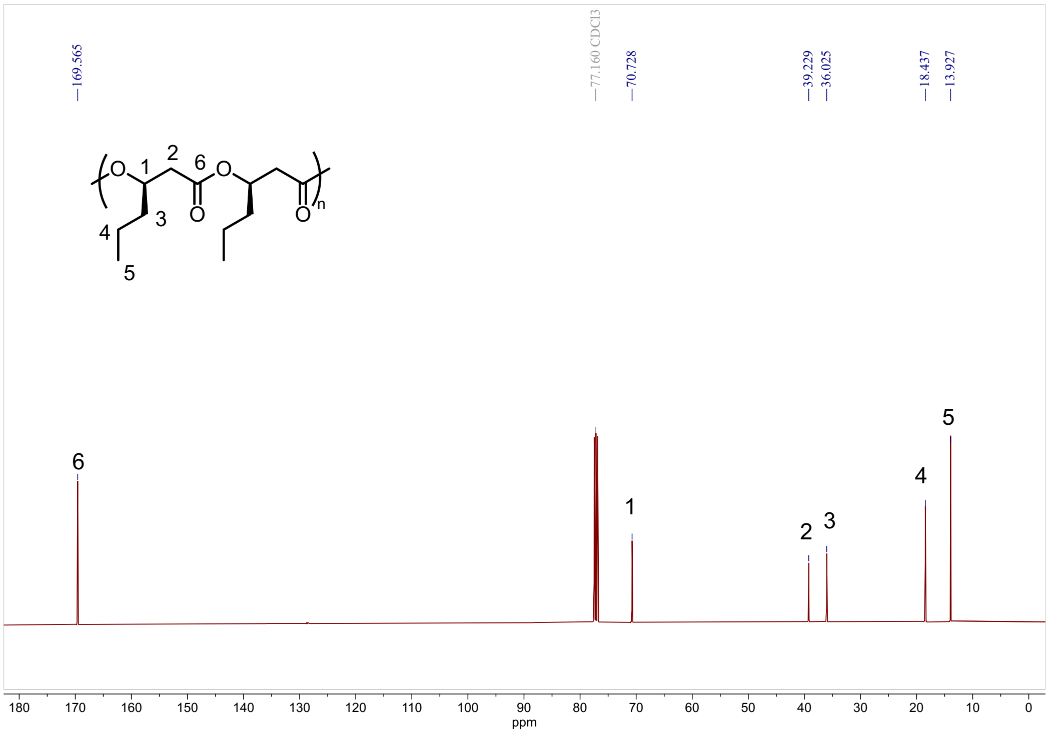

Figure S6. ^{13}C NMR ($CDCl_3$, 23 °C) of *it*-P3HHx.

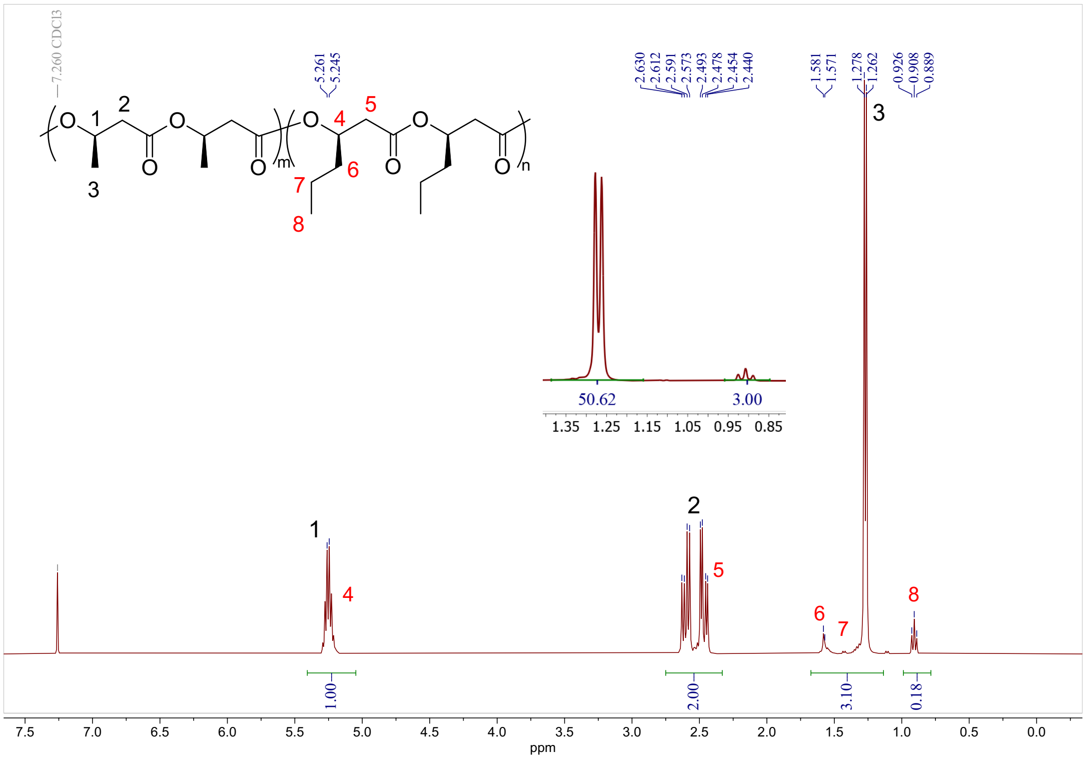

Figure S7. ^{1}H NMR ($CDCl_3$, 23 °C) of statistical copolymer P3HBHx (5.9% 3HHx incorporation).

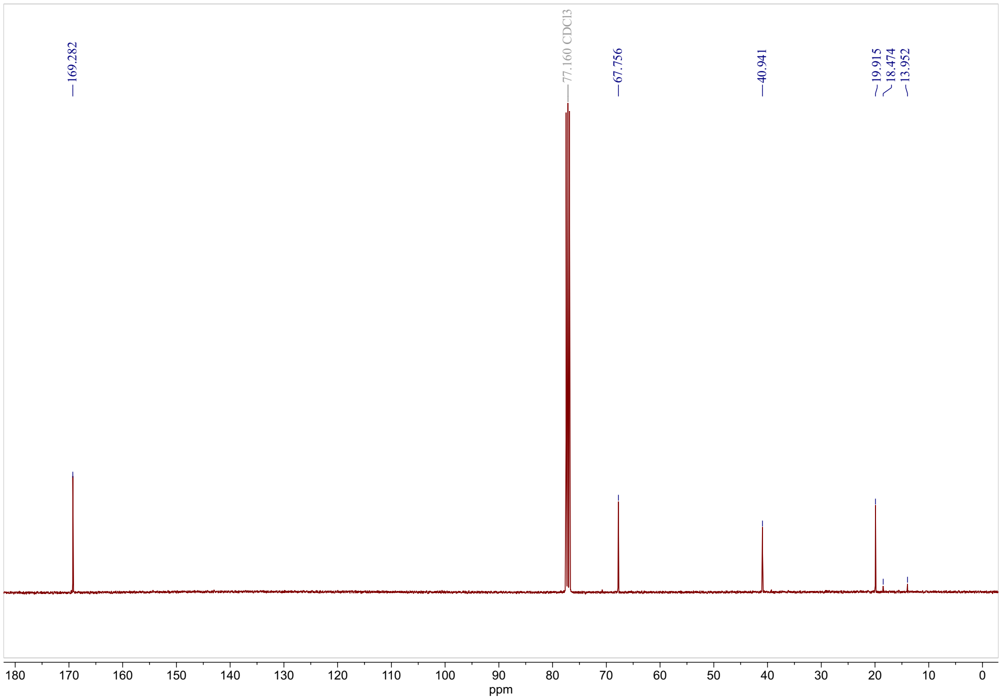

Figure S8. ^{13}C NMR ($CDCl_3$, 23 °C) of statistical copolymer P3HBHx (5.9% 3HHx incorporation).

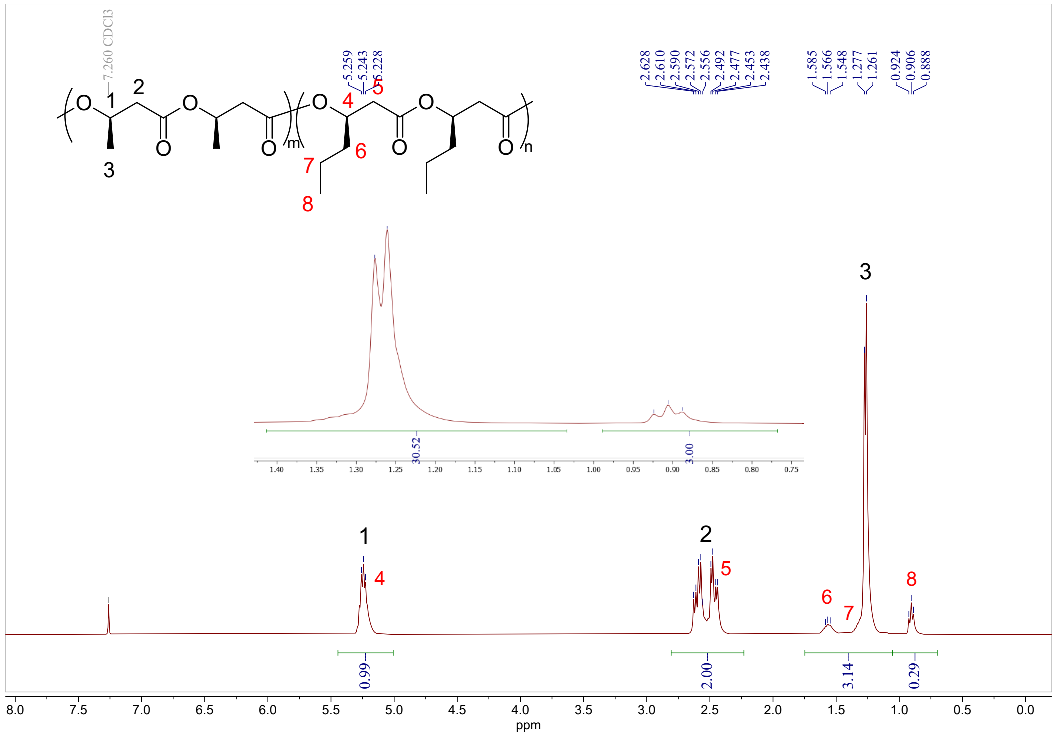

Figure S9. ^{1}H NMR ($CDCl_3$, 23 °C) of statistical copolymer P3HBHx (9.2% 3HHx incorporation).

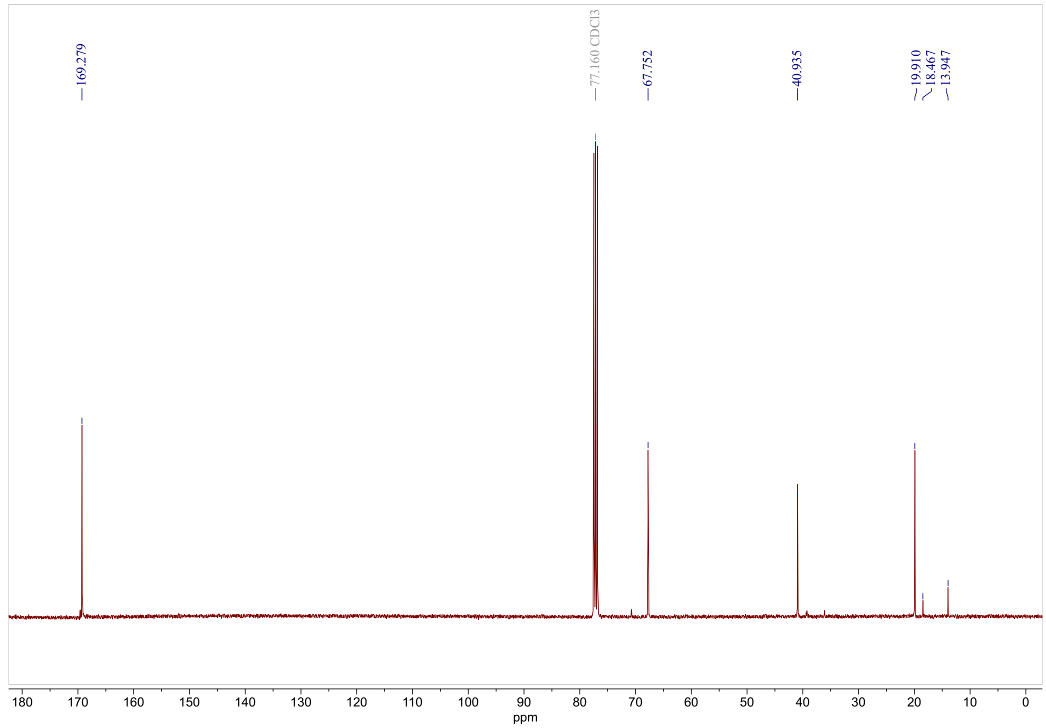

Figure S10. ^{13}C NMR ($CDCl_3$, 23 °C) of statistical copolymer P3HBHx (9.2% 3HHx incorporation).

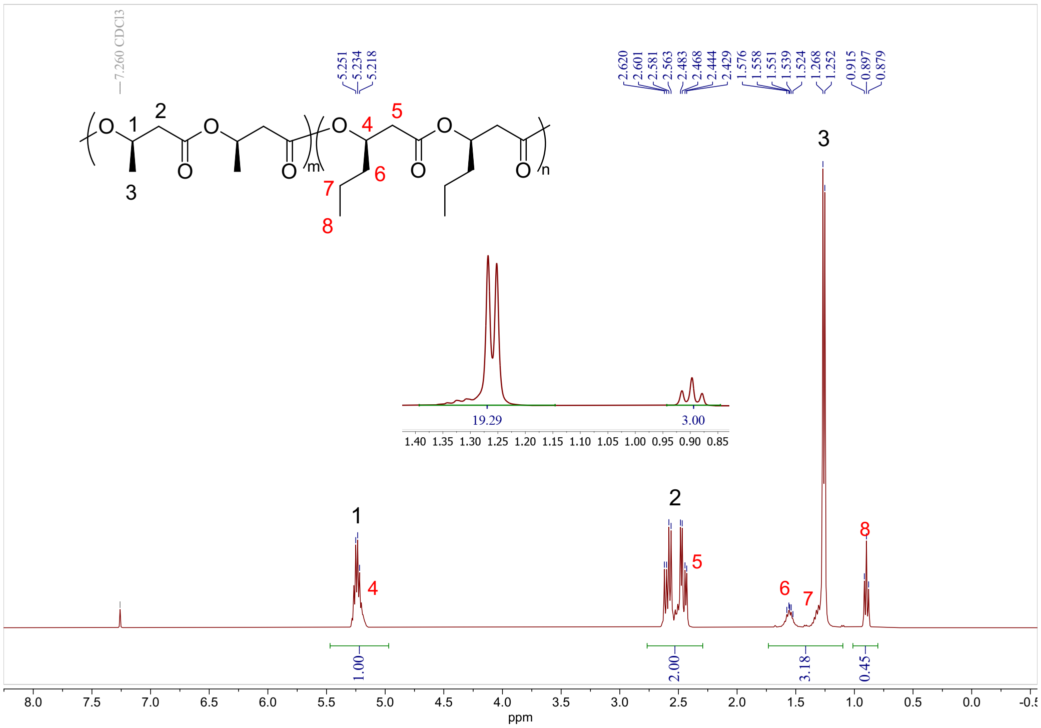

Figure S11. ^{1}H NMR ($CDCl_3$, 23 °C) of statistical copolymer P3HBHx (14.7% 3HHx incorporation).

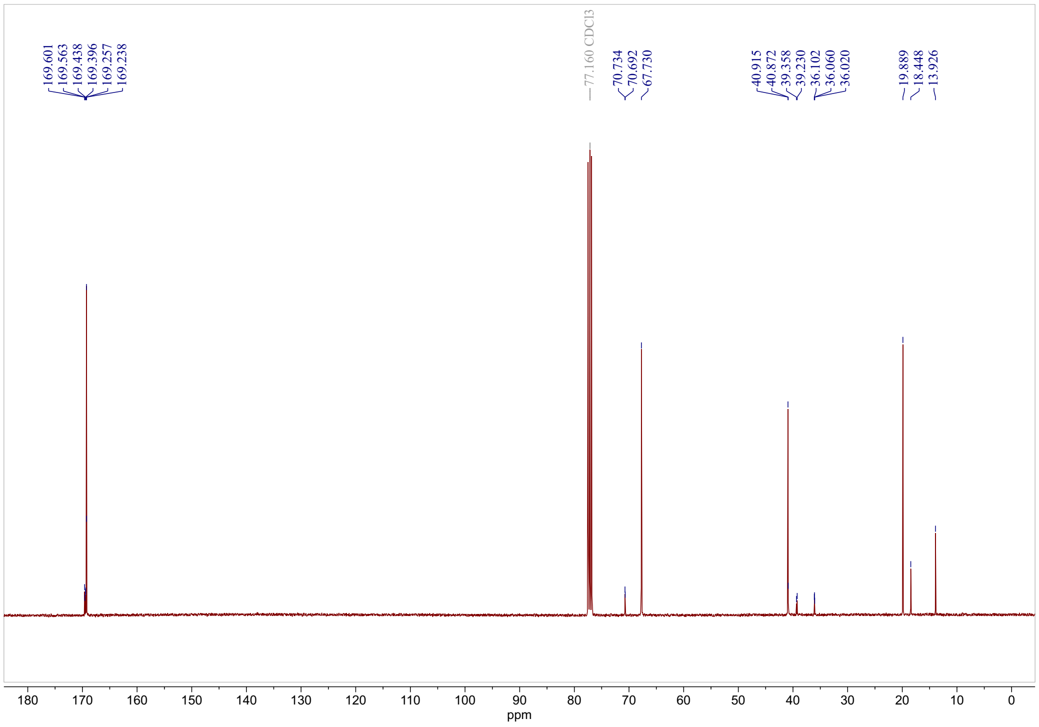

Figure S12. ^{13}C NMR ($CDCl_3$, 23 °C) of statistical copolymer P3HBHx (14.7% 3HHx incorporation).

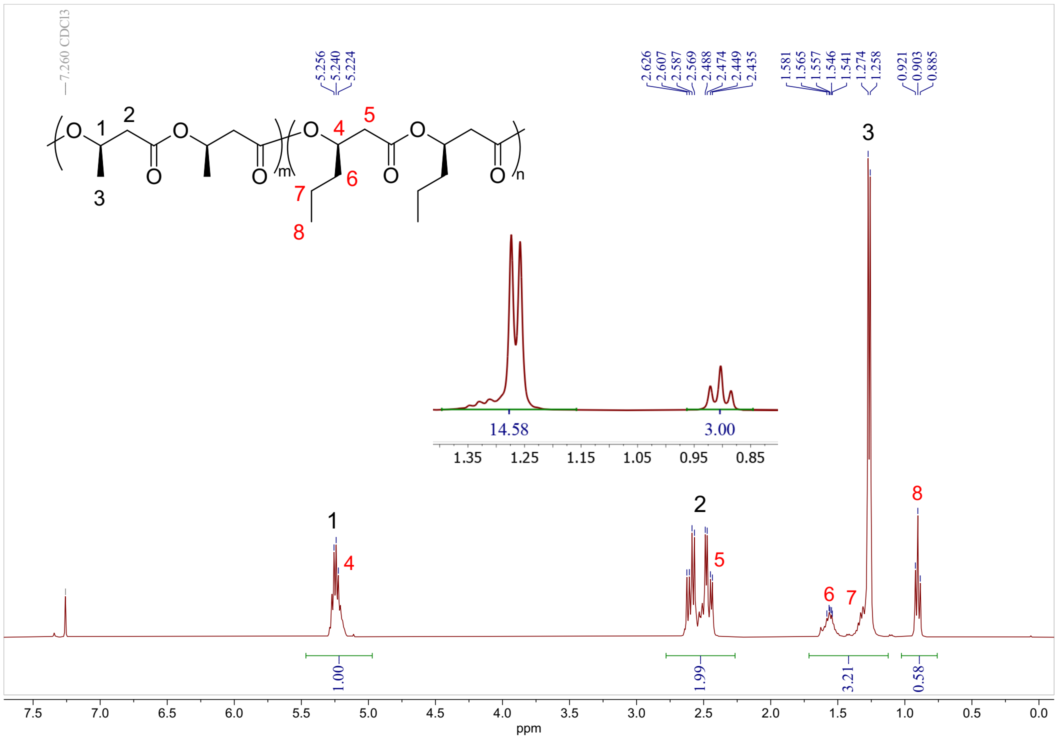

Figure S13. ^{1}H NMR ($CDCl_3$, 23 °C) of statistical copolymer P3HBHx (19% 3HHx incorporation).

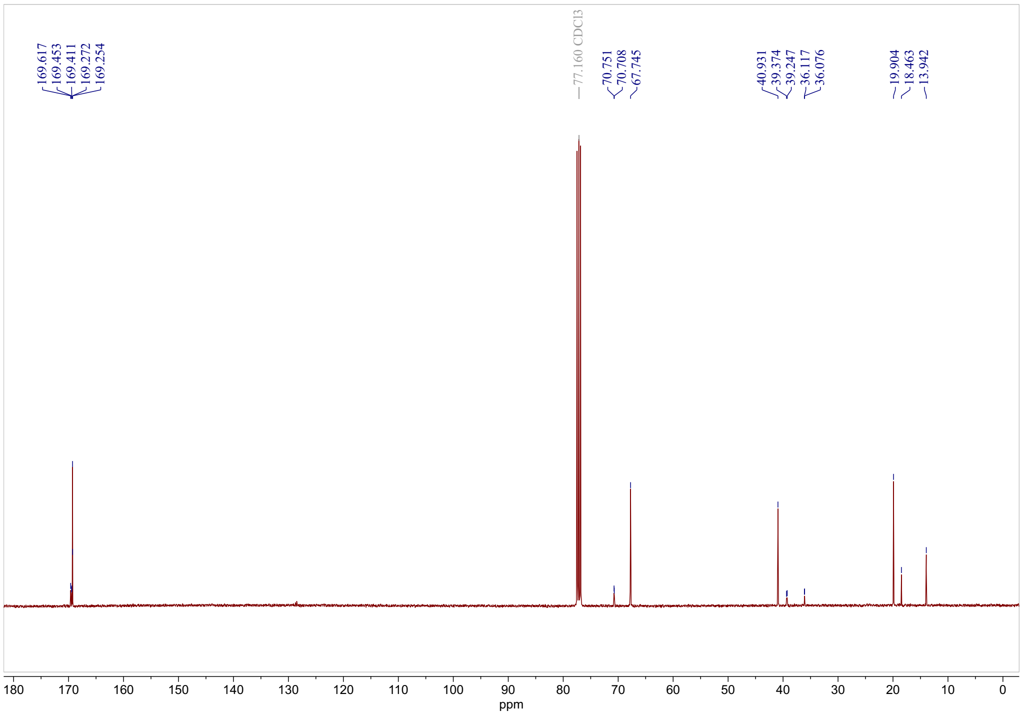

Figure S14. ^{13}C NMR ($CDCl_3$, 23 °C) of statistical copolymer P3HBHx (19% 3HHx incorporation).

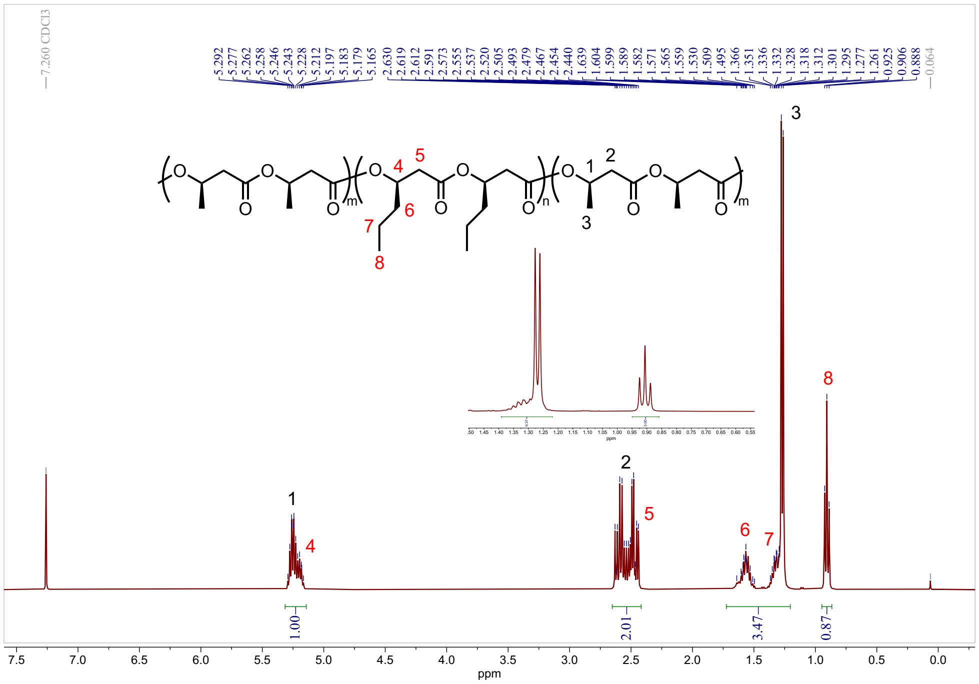

Figure S15. ^{1}H NMR ($CDCl_3$, 23 °C) of triblock copolymer P3HB-*b*-P3HHx-*b*-P3HB (28.9% 3HHx midblock incorporation).

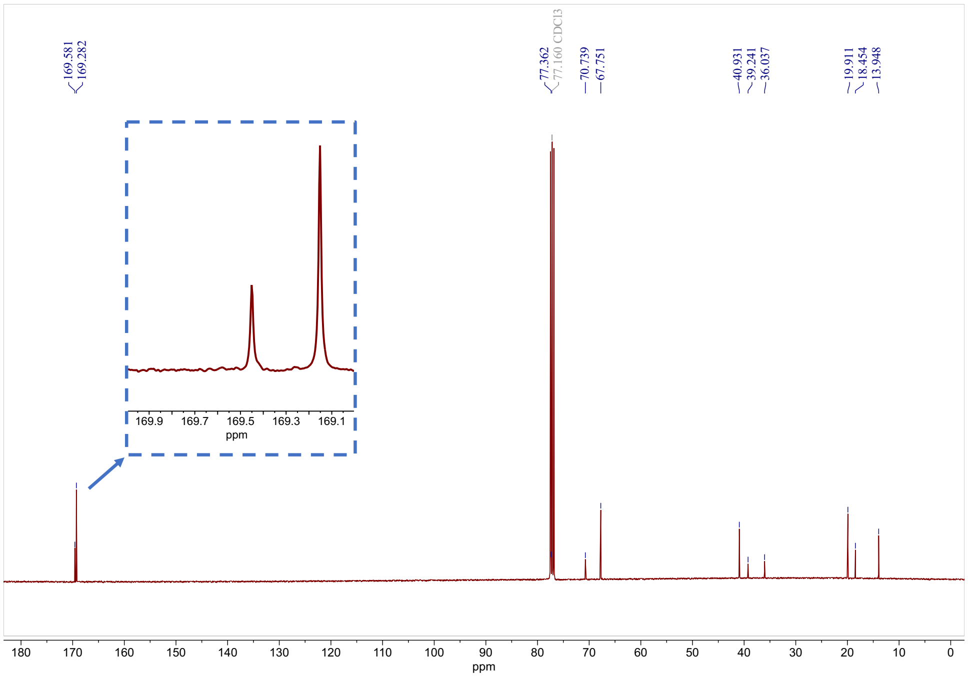

Figure S16. ^{13}C NMR ($CDCl_3$, 23 °C) of triblock copolymer P3HB-*b*-P3HHx-*b*-P3HB (28.9% 3HHx midblock incorporation).

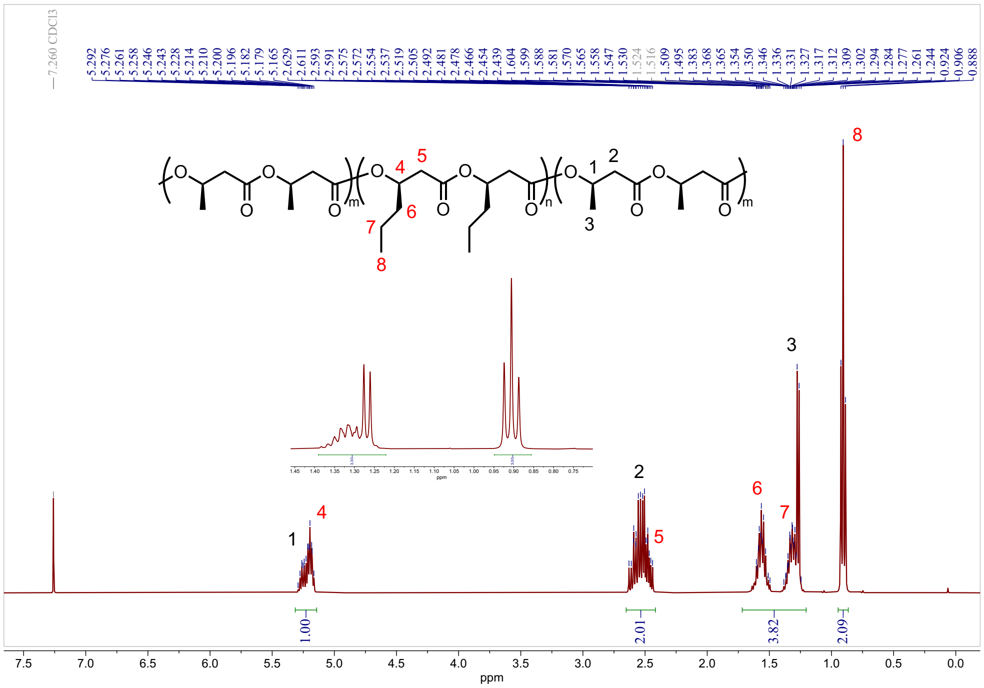

Figure S17. ^{1}H NMR ($CDCl_3$, 23 °C) of triblock copolymer P3HB-*b*-P3HHx-*b*-P3HB (69.8% 3HHx midblock incorporation).

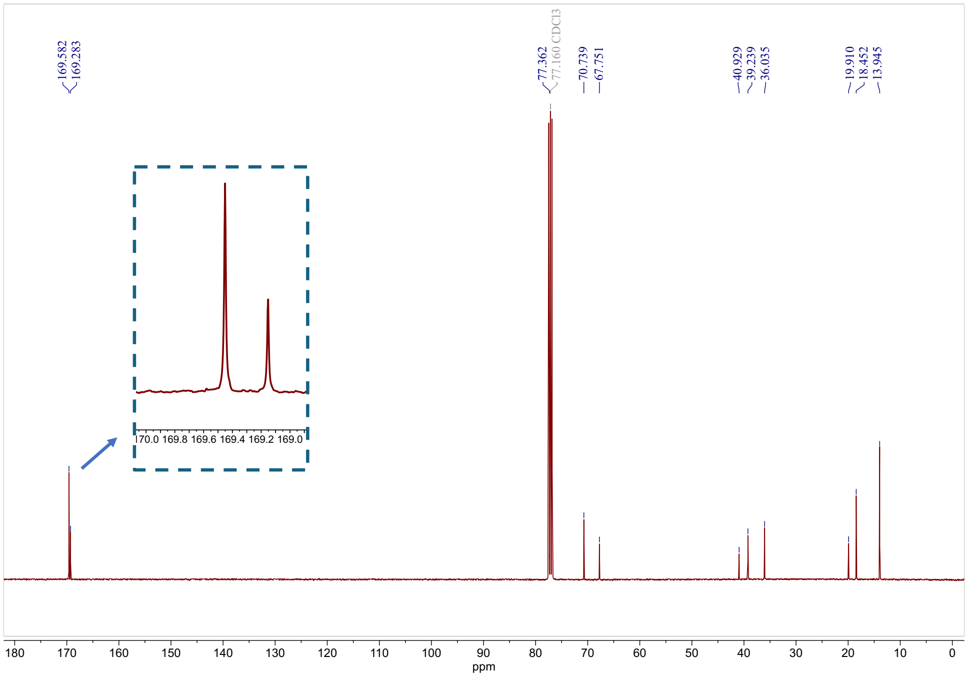

Figure S18. ^{13}C NMR ($CDCl_3$, 23 °C) of triblock copolymer P3HB-*b*-P3HHx-*b*-P3HB (69.8% 3HHx midblock incorporation).

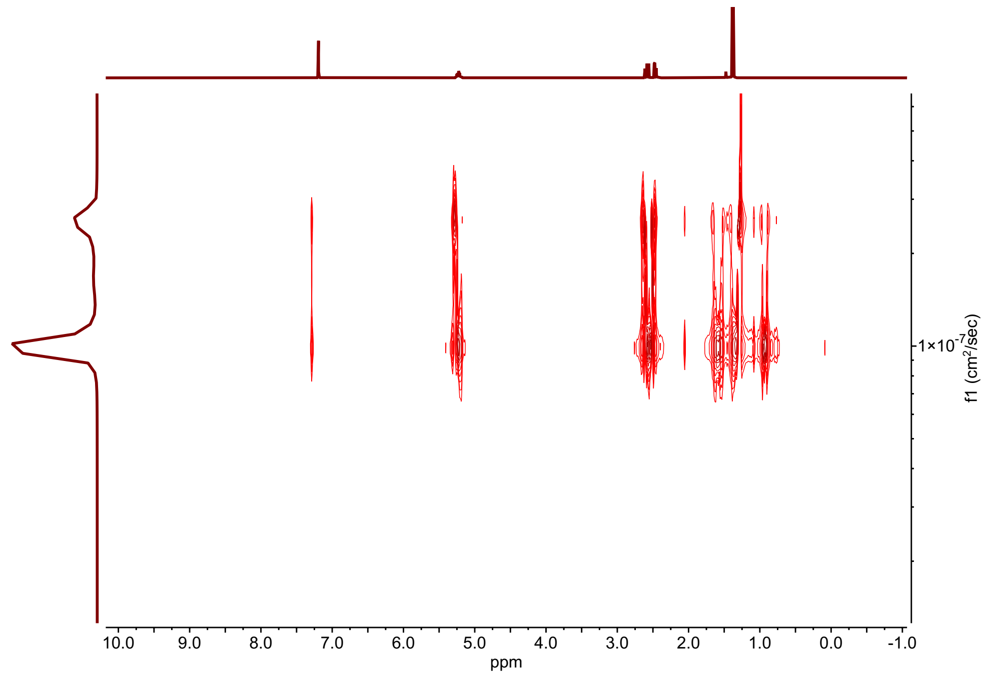

Figure S19. DOSY spectrum ($CDCl_3$, 23 °C) of a binary blend (2:1) mixture of P3HHx and P3HB homopolymers.

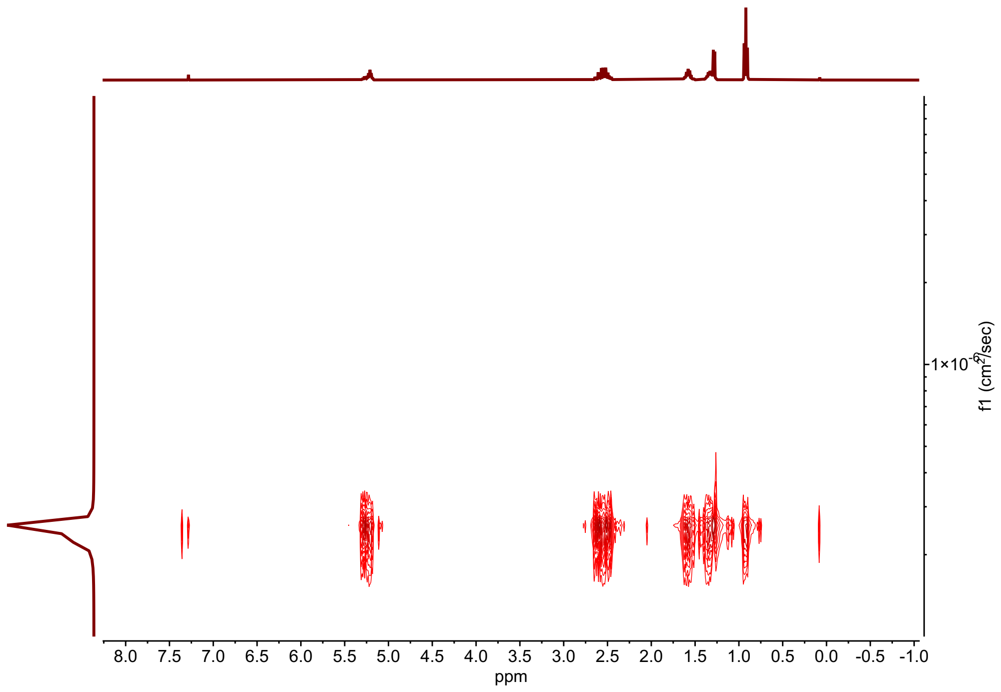

Figure S20. DOSY spectrum ($CDCl_3$, 23 °C) of P3HB-*b*-P3HHx-*b*-P3HB (Run 8, Table 2).

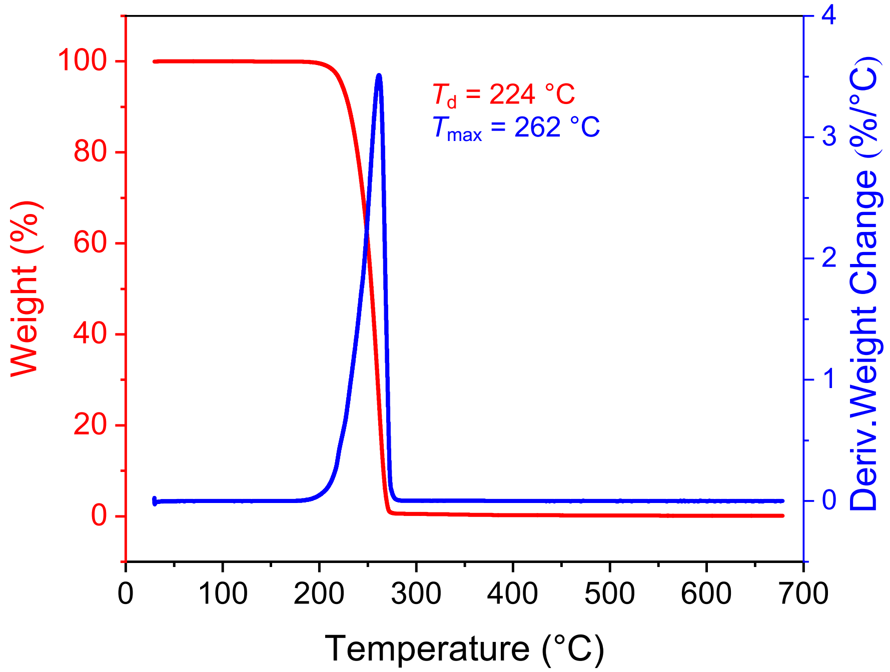

Figure S21. TGA curve of homopolymer *it*-P3HBHx (M_n = 501 kg mol^{-1}, $Đ$ = 1.31, P_m = 0.98).

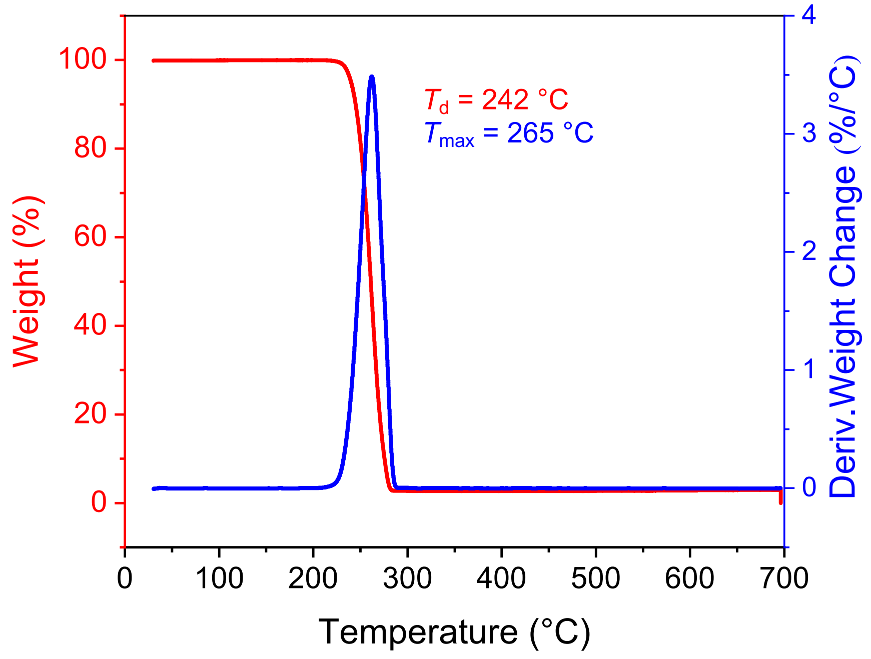

Figure S22. TGA curve of statistical copolymer P3HBHx (M_n = 551 kg mol^{-1}, $Đ$ = 1.29, 5.9% incorporation of 3HHx units).

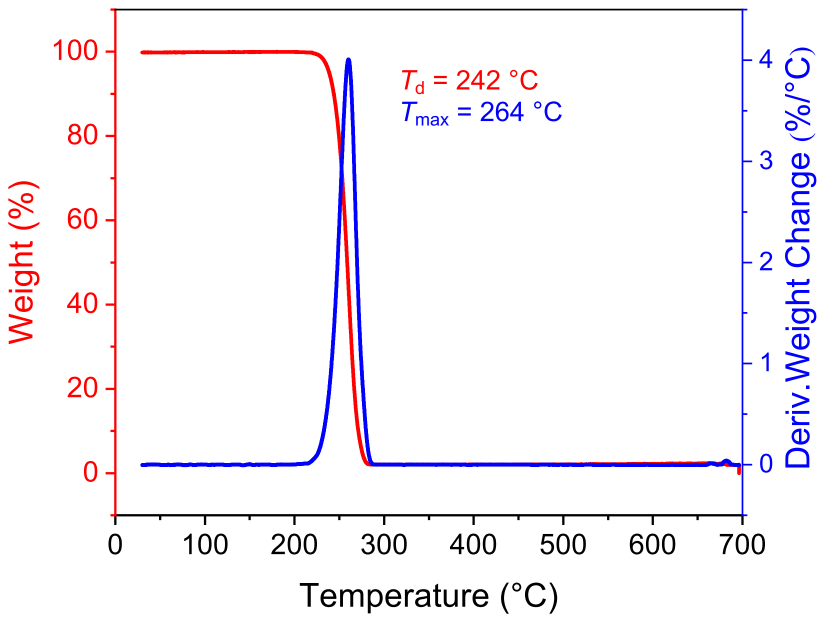

Figure S23. TGA curve of statistical copolymer P3HBHx (M_n = 368 kg mol^{-1}, $Đ$ = 1.13, 14.7% incorporation of 3HHx units).

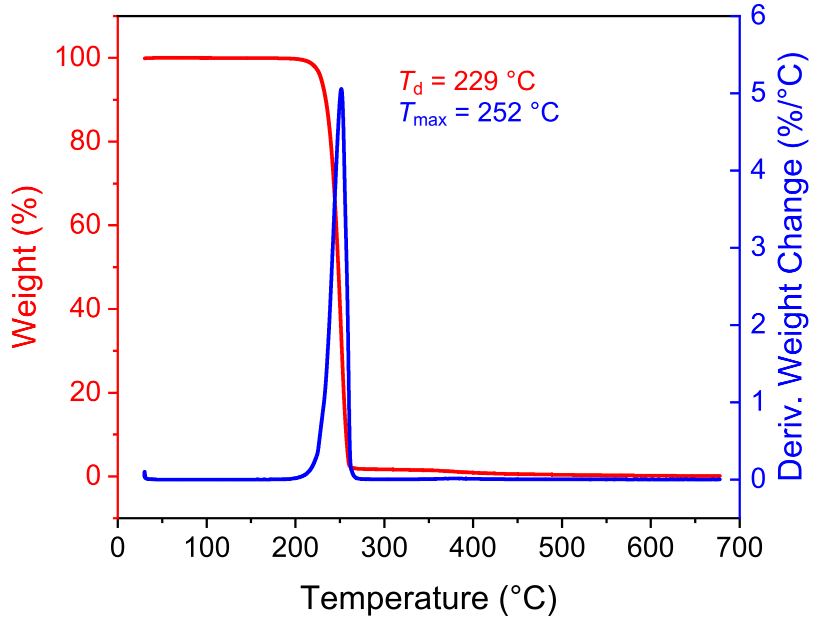

Figure S24. TGA curve of triblock copolymer P3HB-*b*-P3HHx-*b*-P3HB (M_n = 163 kg mol^{-1}, $Đ$ = 1.23, 28.9% incorporation of 3HHx units).

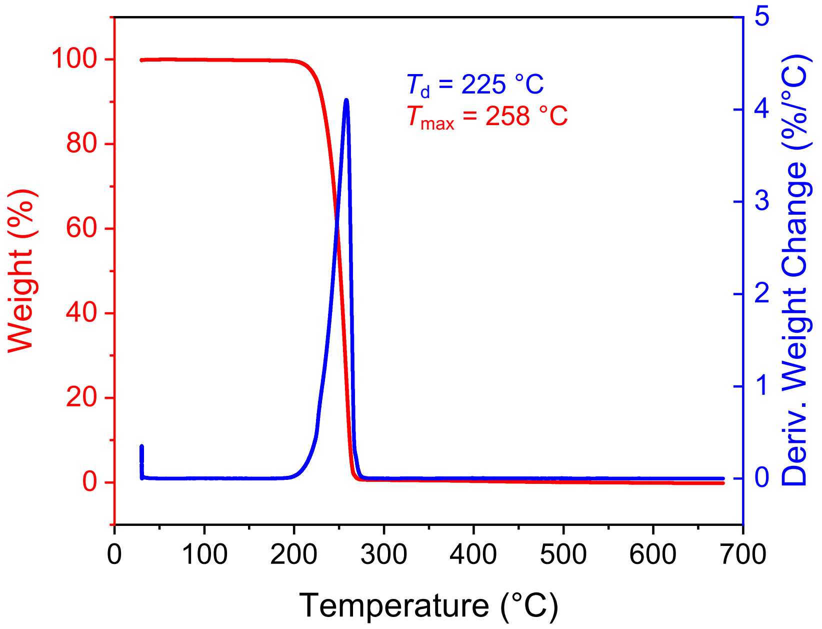

Figure S25. TGA curve of triblock copolymer P3HB-*b*-P3HHx-*b*-P3HB (M_n = 168 kg mol^{-1}, $Đ$ = 1.28, 69.8% incorporation of 3HHx units).

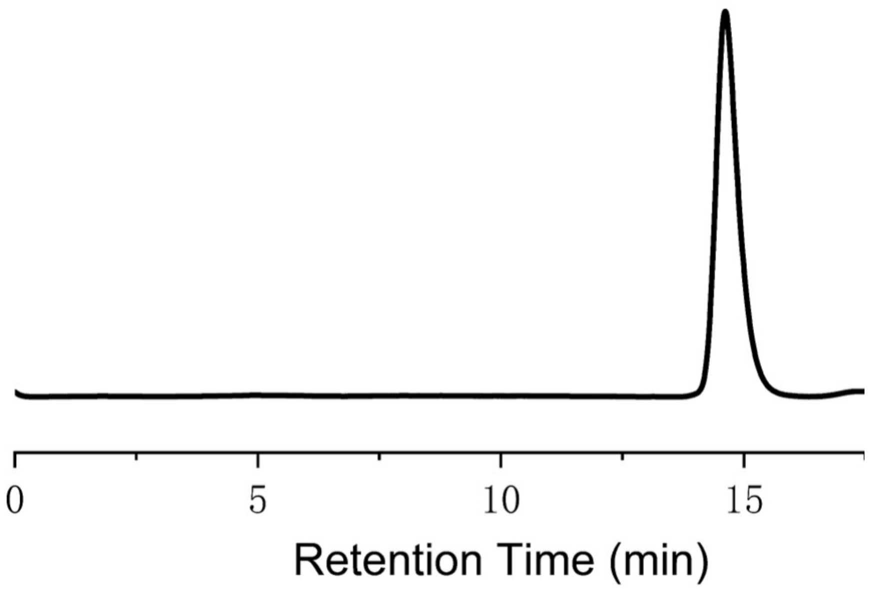

Figure S26. SEC trace of *it*-P3HHx (M_n = 60.5 kg mol^{-1}, $Đ$ = 1.03) (Run 1, Table 1).

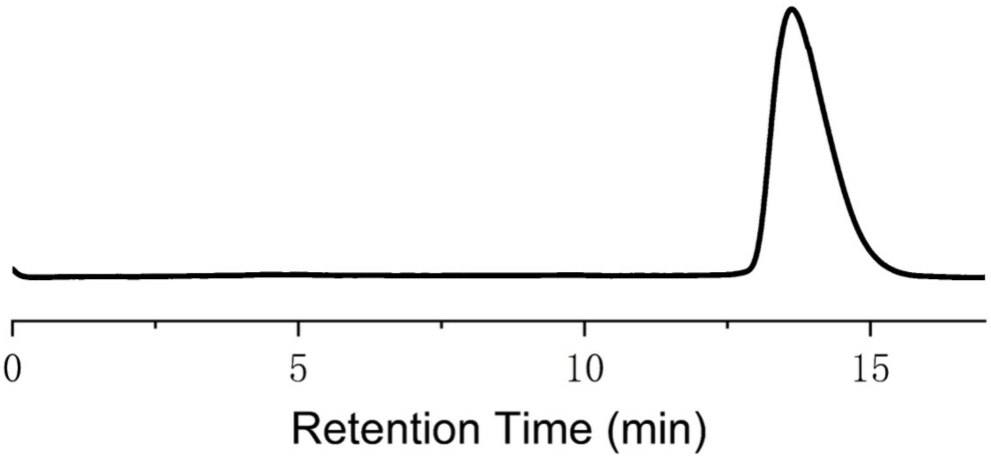

Figure S27. SEC trace of *st*-P3HHx (M_n = 38.1 kg mol^{-1}, $Đ$ = 1.29) (Run 4, Table 1).

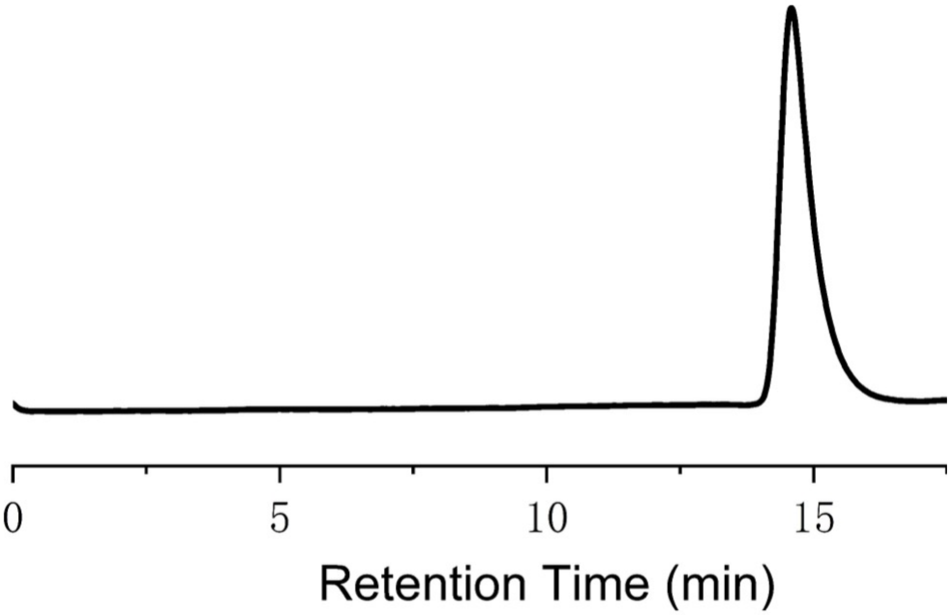

Figure S28. SEC trace of statistical copolymer P3HBHx (M_n = 65 kg mol^{-1}, $Đ$ = 1.10, 6.2% incorporation of 3HHx units) (Run 1, Table 2).

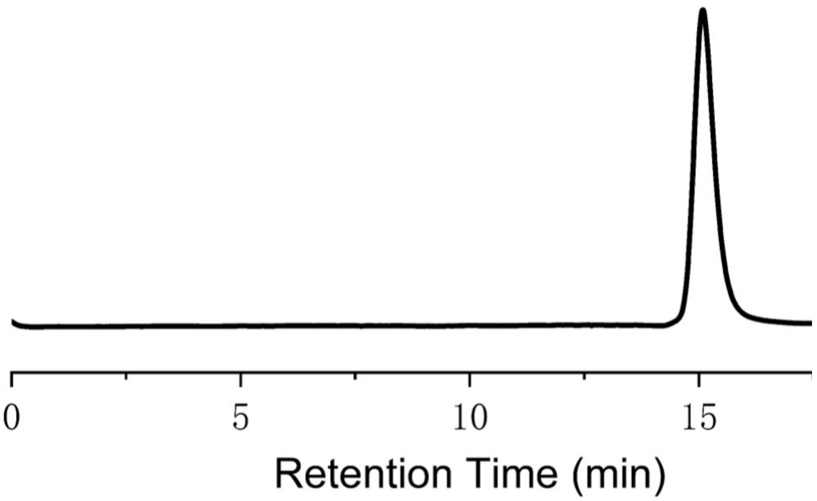

Figure S29. SEC trace of statistical copolymer P3HBHx (M_n = 27.9 kg mol^{-1}, $Đ$ = 1.08, 9.5% incorporation of 3HHx units) (Run 3, Table 2).

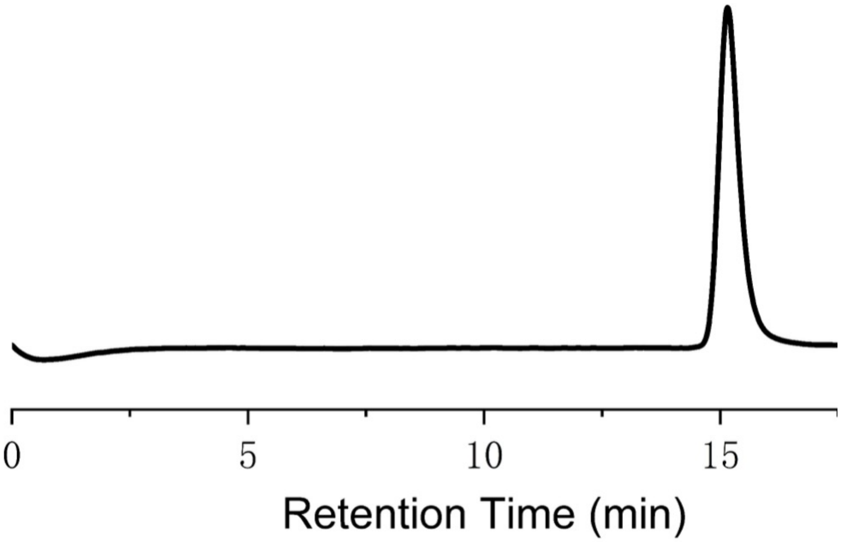

Figure S30. SEC trace of statistical copolymer P3HBHx (M_n = 25.0 kg mol^{-1}, $Đ$ = 1.02, 19% incorporation of 3HHx units) (Run 5, Table 2).

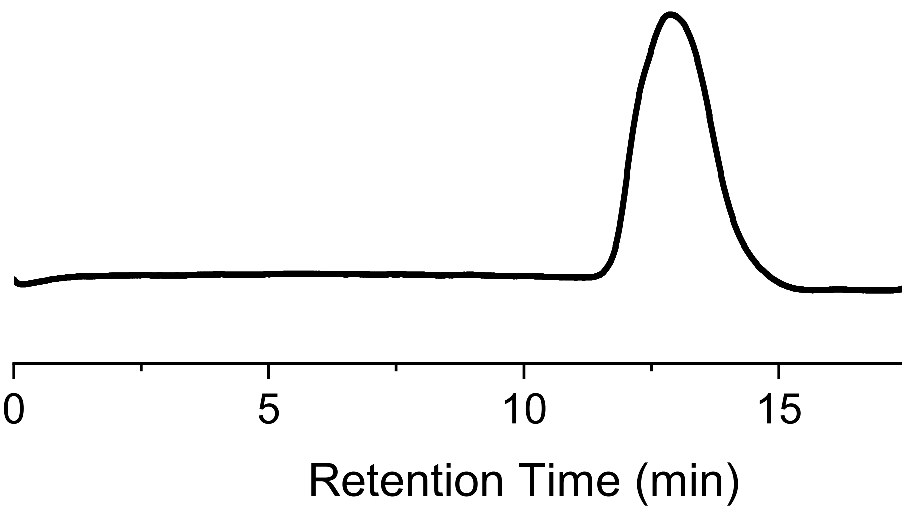

Figure S31. SEC trace of triblock copolymer P3HB-*b*-P3HHx-*b*-P3HB (M_n = 163 kg mol^{-1}, $Đ$ = 1.23, 28.9% incorporation of 3HHx units) (Run 7, Table 2).

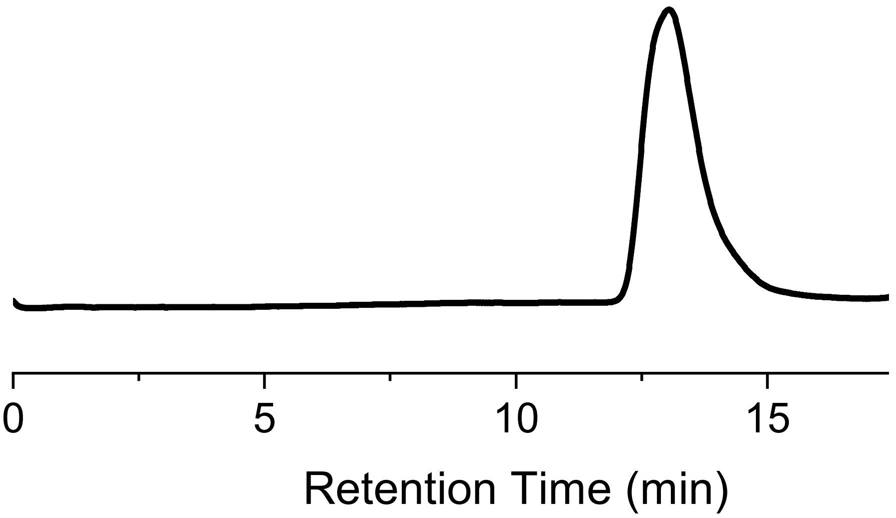

Figure S32. SEC trace of triblock copolymer P3HB-*b*-P3HHx-*b*-P3HB (M_n = 168 kg mol^{-1}, $Đ$ = 1.28, 69.8% incorporation of 3HHx units) (Run 8, Table 2).

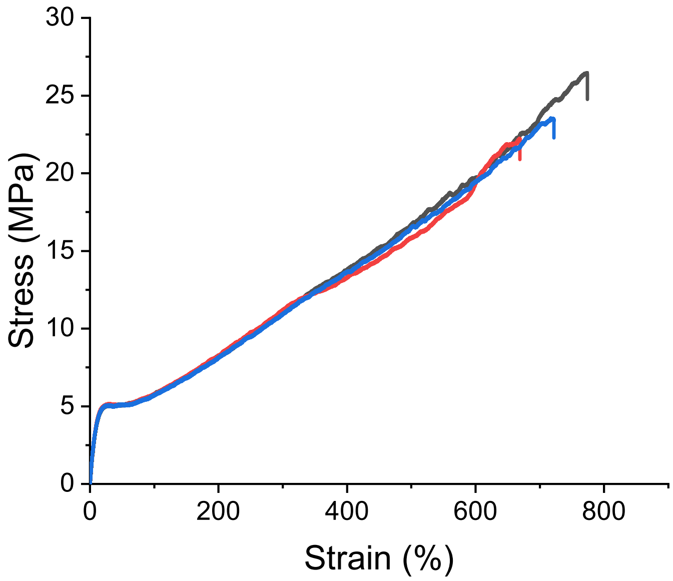

Figure S33. Stress-strain curves of statistical copolymer P3HBHx copolymerized by *meso*-8DL^{Me} and *rac*-8DL^{Pr} (incorporation of 3HHx = 15.2%, M_n = 390.1 kg mol^{-1}, $Đ$ = 1.23).

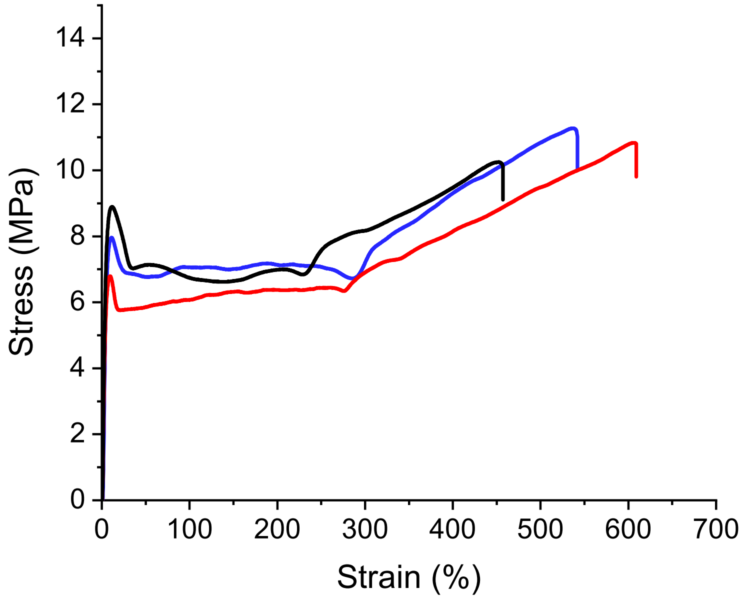

Figure S34. Stress-strain curves of triblock copolymer P3HB-*b*-P3HHx-*b*-P3HB (28.9% incorporation of 3HHx units) (Run 7, Table 2).

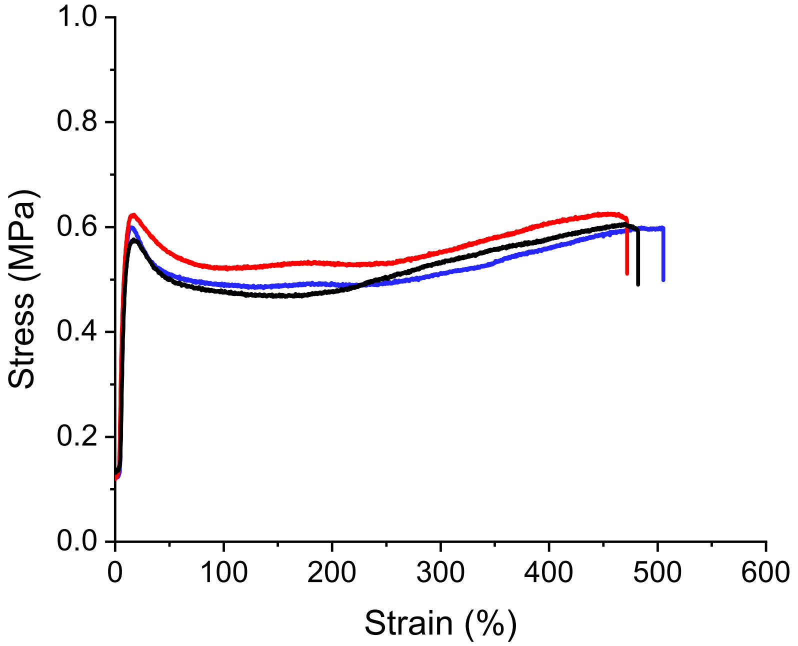

Figure S35. Stress-strain curves of triblock copolymer P3HB-*b*-P3HHx-*b*-P3HB (69.8% incorporation of 3HHx units) (Run 8, Table 2).

Additional Tables

Table S1. Measured tensile behavior of P3HBHx copolymerized by *rac*-8DL^{Me} and *rac*-8DL^{Pr} (3HHx incorporation = 5.9%, M_n = 551 kg mol^{-1}, $Đ$ = 1.29) dog-bone shaped specimens (ASTM D638-5).

Specimen	Modulus of Elasticity (Young's Modulus) (MPa)	Tensile Strength (MPa)	% Elongation at Break	Toughness (MJ m^{-3})
1	1300	23.3	41.3	4.3
2	1540	23.7	18.0	2.8
3	1340	22.6	49.6	4.9
Mean	1390 ± 127	23.2 ± 0.5	36.3 ± 13	4 ± 0.8

Table S2. Measured tensile behavior of P3HBHx copolymerized by *rac*-8DL^{Me} and *rac*-8DL^{Pr} (3HHx incorporation = 9.2%, M_n = 444 kg mol^{-1}, $Đ$ = 1.19) dog-bone shaped specimens (ASTM D638-5).

Specimen	Modulus of Elasticity (Young's Modulus) (MPa)	Tensile Strength (MPa)	% Elongation at Break	Toughness (MJ m^{-3})
1	945	12.2	316	23.6
2	974	14.5	258	24.4
3	927	18.6	372	47.7
Mean	949 ± 19	15 ± 2.6	315 ± 47	31.9 ± 11

Table S3. Measured tensile behavior of P3HBHx copolymerized by *rac*-8DL^{Me} and *rac*-8DL^{Pr} (3HHx incorporation = 14.7%, M_n = 368 kg mol^{-1}, $Đ$ = 1.13) dog-bone shaped specimens (ASTM D638-5).

Specimen	Modulus of Elasticity (Young's Modulus) (MPa)	Tensile Strength (MPa)	% Elongation at Break	Toughness (MJ m^{-3})
1	486	16.6	445	49.1
2	506	15.0	371	31.2
3	498	19.7	520	61.0
Mean	497 ± 8.2	17.1 ± 1.9	445 ± 60	47.1 ± 12

Table S4. Measured tensile behavior of P3HBHx copolymerized by *meso*-8DL^{Me} and *rac*-8DL^{Pr} (3HHx incorporation = 15.2%, M_n = 390.1 kg mol^{-1}, $Đ$ = 1.23) dog-bone shaped specimens (ASTM D638-5).

Specimen	Modulus of Elasticity (Young's Modulus) (MPa)	Tensile Strength (MPa)	% Elongation at Break	Toughness (MJ m^{-3})
1	22.9	26.5	781	80.2
2	35.5	22.2	675	92.6
3	31.3	23.5	723	107
Mean	29.9 ± 5.24	24.1 ± 1.80	726 ± 43.3	93.2 ± 10.9

Table S5. Measured tensile behavior of triblock copolymer P3HB-*b*-P3HHx-*b*-P3HB copolymerized by *rac*-8DL^{Me} and *rac*-8DL^{Pr} (3HHx incorporation = 28.9%, M_n = 163 kg mol^{-1}, $Đ$ = 1.23) dog-bone shaped specimens (ASTM D638-5).

Specimen	Modulus of Elasticity (Young's Modulus) (MPa)	Tensile Strength (MPa)	% Elongation at Break	Toughness (MJ m^{-3})
1	400	11.0	541	43.9
2	388	10.8	609	45.9
3	426	10.1	457	35.9
Mean	405 ± 19	10.6 ± 0.5	536 ± 76	41.9 ± 5.3

Table S6. Measured tensile behavior of triblock copolymer P3HB-*b*-P3HHx-*b*-P3HB copolymerized by *rac*-8DL^{Me} and *rac*-8DL^{Pr} (3HHx incorporation = 69.8%, M_n = 168 kg mol^{-1}, $Đ$ = 1.29) dog-bone shaped specimens (ASTM D638-5).

Specimen	Modulus of Elasticity (Young's Modulus) (MPa)	Tensile Strength (MPa)	% Elongation at Break	Toughness (MJ m^{-3})
1	5.3	0.60	501	2.63
2	5.6	0.61	472	2.62
3	5.2	0.59	482	2.50
Mean	5.4 ± 0.20	0.60 ± 0.01	486 ± 17	2.6 ± 0.07
